# Tumor microenvironment-driven therapeutic resistance in pancreatic ductal adenocarcinoma

**DOI:** 10.20517/cdr.2026.102

**Published:** 2026-10-19

**Authors:** Hong-Jia Li, Jia-Ning Cui, Xin Yang, Jia-Hang Li, Qin-Cheng Zhao, Chun-Jie Li, Qiang Fu, Jun-Ying Li, Yu Zhou, Shi-Kai Zhu

**Affiliations:** ^1^Organ Transplant Center, Sichuan Provincial People’s Hospital, School of Medicine, University of Electronic Science and Technology of China, Chengdu 610072, Sichuan, China.; ^2^Translational Clinical Immunology Key Laboratory of Sichuan Province, Sichuan Provincial People’s Hospital, School of Medicine, University of Electronic Science and Technology of China, Chengdu 610072, Sichuan, China.; ^3^Emergency Medicine Center, Sichuan Provincial People’s Hospital, School of Medicine, University of Electronic Science and Technology of China, Chengdu 610072, Sichuan, China.; ^4^Genetic Diseases Key Laboratory of Sichuan Province, Center for Medical Genetics, Sichuan Provincial People’s Hospital, School of Medicine, University of Electronic Science and Technology of China, Chengdu 610072, Sichuan, China.; ^#^These authors equally contributed to this work.

**Keywords:** Pancreatic ductal adenocarcinoma, tumor microenvironment, therapeutic resistance, stromal remodeling, metabolic-immune crosstalk, precision therapy

## Abstract

Pancreatic ductal adenocarcinoma (PDAC) remains one of the most treatment-refractory malignancies, with limited and often transient benefit from chemotherapy, targeted therapy, immunotherapy, and radiotherapy. Therapeutic resistance is increasingly recognized as an ecosystem-level process arising from reciprocal interactions between malignant cells and the tumor microenvironment (TME). Dense extracellular matrix, cancer-associated fibroblasts (CAFs), pancreatic stellate cells (PSCs), dysfunctional vasculature, immunosuppressive myeloid and lymphoid populations, hypoxic niches, metabolic networks, microbiota, extracellular vesicles (EVs), and neural components collectively restrict drug delivery and promote adaptive survival, immune escape, stemness, and treatment-induced evolution. This review integrates these processes into a PDAC resistance ecosystem framework. We discuss how stromal remodeling, hypoxia-driven adaptation, metabolic-immune crosstalk, extracellular communication, cancer stem cell plasticity, and TME-regulated autophagy and ferroptosis contribute to resistance across therapeutic modalities. We further evaluate emerging strategies involving stromal reprogramming, myeloid and immune remodeling, metabolic intervention, hypoxia targeting, and regulated cell-death sensitization. Particular emphasis is placed on the limited clinical translation of indiscriminate stromal depletion and single-target approaches. Finally, we propose that spatial multi-omics, longitudinal biomarker profiling, and adaptive combination therapy will be required to identify dominant resistance programs and guide patient-specific intervention in PDAC.

## INTRODUCTION

Pancreatic ductal adenocarcinoma (PDAC) is one of the most aggressive and lethal solid malignancies, largely because of late diagnosis, early metastatic dissemination, and profound therapeutic resistance^[1-3]^. Although surgical resection remains the only potentially curative treatment, more than 80% of patients present with locally advanced or metastatic disease, leaving systemic therapy as the principal treatment option for most patients^[4]^. Chemotherapy remains the backbone of treatment, but the benefits of current regimens, including gemcitabine plus nab-paclitaxel and FOLFIRINOX, are substantially limited by intrinsic and acquired resistance^[5,6]^. Molecularly targeted and immune-based therapies have expanded therapeutic opportunities in selected settings; however, durable responses remain uncommon in unselected PDAC, highlighting the need to understand resistance mechanisms beyond tumor-cell-intrinsic alterations^[7,8]^.

Accumulating evidence indicates that therapeutic resistance in PDAC cannot be explained solely by tumor-cell-intrinsic alterations. The uniquely desmoplastic, hypovascular, hypoxic, and immunosuppressive tumor microenvironment (TME) actively regulates therapeutic response through interconnected physical, cellular, metabolic, and signaling mechanisms^[9-12]^. Dense extracellular matrix (ECM) deposition and stromal remodeling compress tumor vasculature and restrict drug delivery; cancer-associated fibroblast (CAF)-derived signals activate pro-survival pathways; hypoxia and metabolic competition promote adaptive survival; and immunosuppressive myeloid and lymphoid populations constrain antitumor immunity^[13-16]^. Importantly, these mechanisms do not operate independently but interact dynamically with malignant cells and can be further reshaped by therapeutic pressure, thereby generating both intrinsic and adaptive resistance.

Although stromal, immune, hypoxic, and metabolic mechanisms of PDAC resistance have been extensively studied, they are often considered as separate processes. Recent single-cell and spatial profiling studies have instead revealed spatially organized and functionally interconnected stromal, immune, and metabolic niches, indicating that therapeutic resistance emerges from coordinated interactions among multiple tumor and microenvironmental compartments^[15,17-19]^. Moreover, therapeutic pressure can remodel these interactions, promoting adaptive signaling, metabolic rewiring, immune escape, and the selection of resistant cellular states^[20-23]^. We therefore use the term “PDAC resistance ecosystem” to describe a dynamic and spatially organized network in which tumor-intrinsic programs and TME-derived physical, cellular, metabolic, and signaling mechanisms interact and evolve under therapeutic pressure. This framework extends beyond cataloguing individual resistance mechanisms by emphasizing their interdependence, spatial organization, and treatment-induced evolution.

### Scope of the review

Several recent reviews have summarized the PDAC TME, chemotherapy resistance, or emerging TME-targeted therapies^[24,25]^. In contrast, the present review focuses on the TME as a dynamic therapeutic resistance ecosystem, emphasizing reciprocal interactions among stromal, immune, metabolic, hypoxic, neural, microbial, and extracellular vesicle (EV)-associated components and their differential contributions across therapeutic modalities. We further integrate mechanistic evidence with clinical translation and propose an ecosystem-oriented framework for biomarker-guided combination therapy.

### Literature search strategy

A literature search was conducted using PubMed and Web of Science to identify relevant studies on TME-mediated therapeutic resistance in PDAC. Search terms included combinations of “pancreatic ductal adenocarcinoma” or “pancreatic cancer” with “tumor microenvironment”, “therapeutic resistance”, “chemoresistance”, “radiotherapy resistance”, “immunotherapy resistance”, “cancer-associated fibroblasts”, “extracellular matrix”, “immune suppression”, “hypoxia”, “metabolism”, “microbiome”, and “neural signaling”. Priority was given to peer-reviewed original studies, clinical trials, and high-quality reviews relevant to the mechanisms and therapeutic implications discussed herein, with emphasis on recent publications. Studies were excluded when they were not directly relevant to PDAC therapeutic resistance or lacked sufficient mechanistic or clinical relevance. Reference lists of key articles were also screened to identify additional pertinent studies.

## THE PDAC TME AS A THERAPEUTIC RESISTANCE ECOSYSTEM

The TME in PDAC is among the most complex and treatment-refractory microenvironments of all solid malignancies [Figure 1]. Stromal components generate a highly desmoplastic, hypovascular, hypoxic, and immunosuppressive ecosystem that profoundly influences tumor progression, metastatic dissemination, and therapeutic resistance^[9,26-29]^. The PDAC microenvironment consists of both cellular and acellular components. Through extensive bidirectional interactions, these components establish a dynamic ecosystem that promotes tumor survival and treatment resistance^[13,26,27]^.

**Figure 1:**
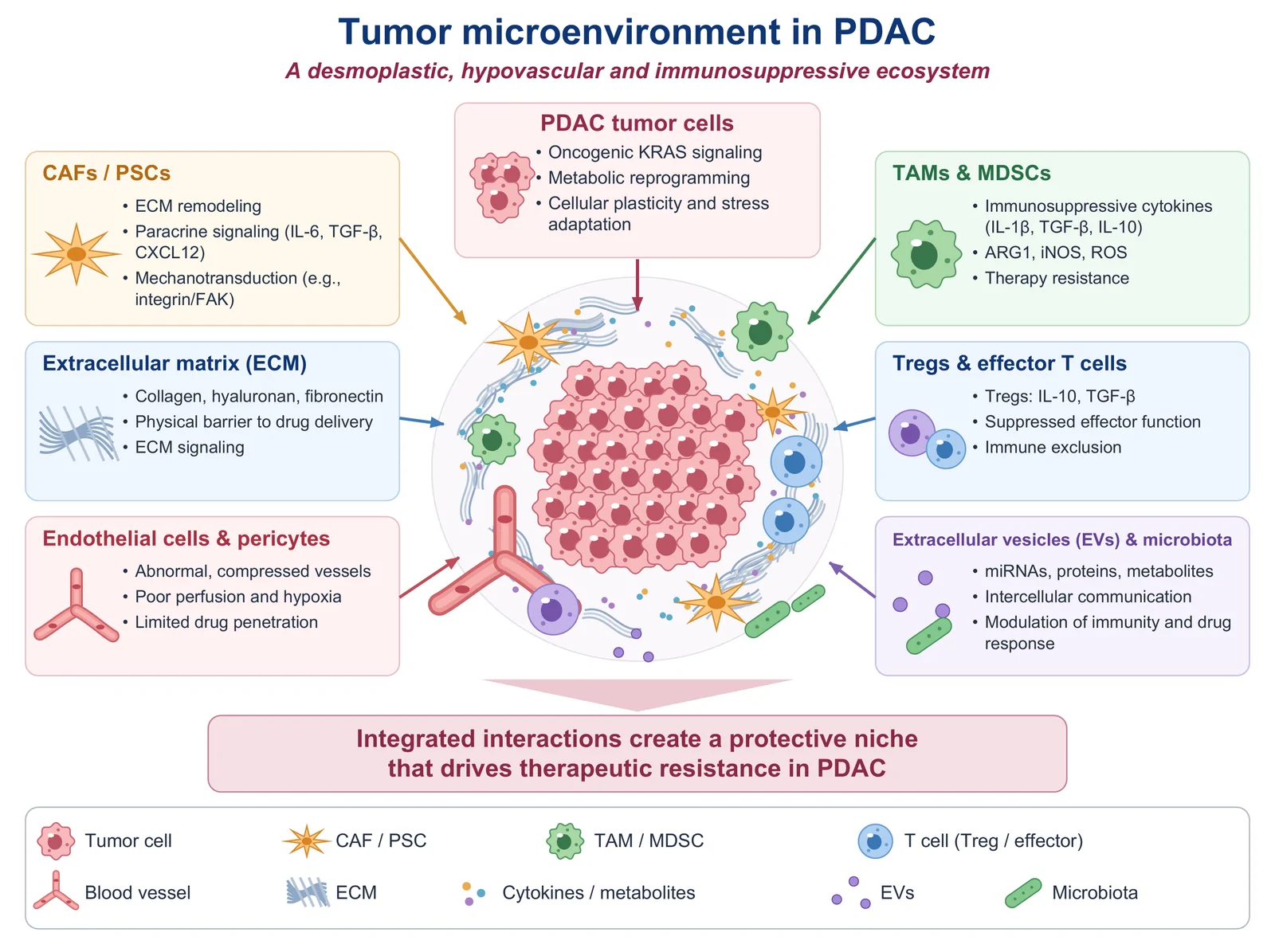
TME in PDAC and its contribution to therapeutic resistance. The PDAC TME comprises malignant cells embedded within a dense and dynamically interacting stromal, immune, vascular, and ECM network. CAFs and PSCs remodel the ECM and provide paracrine and mechanotransductive signals, whereas excessive ECM deposition and abnormal, compressed vasculature impair tumor perfusion and drug penetration. TAMs, MDSCs, and Tregs contribute to an immunosuppressive milieu that limits effective antitumor immunity. EVs, microbiota, cytokines, and metabolites further mediate intercellular communication and modulate therapeutic response. Together with oncogenic signaling, metabolic reprogramming, and cellular plasticity in PDAC cells, these interconnected components establish a protective niche that promotes therapeutic resistance. TME: Tumor microenvironment; PDAC: pancreatic ductal adenocarcinoma; ECM: extracellular matrix; CAFs: cancer-associated fibroblasts; PSCs: pancreatic stellate cells; TAMs: tumor-associated macrophages; MDSCs: myeloid-derived suppressor cells; Tergs: regulatory T cells; EVs: extracellular vesicles; IL-6: interleukin-6; TGF-β: transforming growth factor-β; CXCL12: C-X-C motif chemokine ligand 12; FAK: focal adhesion kinase; KRAS: Kirsten rat sarcoma viral oncogene homolog; IL-1β: interleukin-1 beta; IL-10: interleukin-10; ARG1: arginase 1; iNOS: inducible nitric oxide synthase; ROS: reactive oxygen species; miRNAs: microRNAs.

### Desmoplastic stroma and ECM

The ECM represents one of the most distinctive features of PDAC and is primarily composed of collagen, fibronectin, laminin, hyaluronic acid (HA), proteoglycans, and matricellular proteins^[28-31]^. The excessive deposition of ECM components creates a dense fibrotic network that serves as a primary barrier to drug delivery and infiltration of cytotoxic CD8^+^ T cells^[9,32]^. This desmoplastic stroma increases interstitial fluid pressure, compresses intratumoral blood vessels, and reduces vascular perfusion, thereby limiting drug penetration into tumor tissues^[28,33]^. Consequently, vascular compression and impaired perfusion reduce intratumoral drug exposure, limiting the cytotoxic efficacy of gemcitabine and other systemically administered agents^[33]^. Furthermore, ECM components such as HA and fibronectin may directly bind therapeutic agents, further restricting their diffusion and cellular uptake^[34]^.

Beyond its role as a physical barrier, ECM components actively participate in tumor progression through integrin-mediated signaling pathways. Interactions between ECM proteins and tumor cells activate focal adhesion kinase (FAK), phosphoinositide 3-kinase (PI3K)/protein kinase B (PKB), and mitogen-activated protein kinase (MAPK) signaling cascades, promoting anti-apoptotic signaling, epithelial-mesenchymal transition (EMT), stemness maintenance, and therapeutic resistance^[35]^.

### CAF/pancreatic stellate cell-mediated paracrine and metabolic resistance

Pancreatic stellate cells (PSCs) are the principal producers of ECM within the PDAC microenvironment and are considered central mediators of pancreatic fibrosis^[28,36]^. Under physiological conditions, PSCs remain in a quiescent state characterized by vitamin A-containing lipid droplets. However, during tumor development, PSCs become activated by inflammatory cytokines, oxidative stress, hypoxia, and tumor-derived growth factors, subsequently differentiating into myofibroblast-like cells^[36]^. Activated PSCs secrete large amounts of collagen, fibronectin, and HA, thereby contributing to desmoplasia and increased tissue stiffness^[28]^. In addition to matrix production, PSCs release a variety of cytokines and growth factors, including transforming growth factor-β (TGF-β), hepatocyte growth factor (HGF), vascular endothelial growth factor (VEGF), and interleukin-6 (IL-6), which stimulate tumor proliferation, angiogenesis, invasion, and therapeutic resistance^[11,28]^.

CAFs are among the most abundant stromal cell populations in PDAC and have emerged as key regulators of therapeutic resistance^[20,26,37,38]^. Recent advances in single-cell sequencing have revealed remarkable CAF heterogeneity, overturning the traditional view that CAFs represent a homogeneous population^[39,40]^. Several major CAF subtypes have been identified, including myofibroblastic CAFs (myCAFs), inflammatory CAFs (iCAFs), and antigen-presenting CAFs (apCAFs)^[26,37,38]^. MyCAFs are characterized by high alpha-smooth muscle actin (α-SMA) expression and are primarily involved in ECM deposition and tissue remodeling. In contrast, iCAFs secrete large quantities of inflammatory mediators such as IL-6, C-X-C motif chemokine ligand 12 (CXCL12), and leukemia inhibitory factor (LIF), thereby promoting tumor growth and immune suppression^[20,37]^. ApCAFs express major histocompatibility complex class II (MHC-II) molecules and may participate in antigen presentation and immune modulation^[26]^. LRRC15-positive CAFs represent a TGF-β-dependent myofibroblastic population associated with stromal remodeling, immune exclusion, and poor response to immune checkpoint blockade. In addition, metabolically reprogrammed CAFs can support tumor survival by supplying lactate, amino acids, and lipid metabolites, thereby linking stromal heterogeneity to metabolic adaptation and therapeutic resistance^[38,41-43]^. Thus, CAF-targeted therapy should be guided by functional state and spatial localization rather than total CAF abundance. CAF populations exhibit substantial plasticity. Under the influence of TGF-β, IL-1, and Janus kinase (JAK)/signal transducer and activator of transcription (STAT) signaling pathways, transitions between iCAF and myCAF phenotypes can occur dynamically during tumor progression^[37,38]^.

CAFs and PSCs are major stromal regulators of chemoresistance in PDAC. Through paracrine signaling, EV-mediated communication, and metabolic coupling, these cells actively reprogram tumor behavior and promote survival under therapeutic stress^[28,36,44]^. Activated CAFs and PSCs secrete a broad spectrum of cytokines, chemokines, and growth factors, including IL-6, HGF, TGF-β, CXCL12, and VEGF^[11,12,45]^. Among these mediators, IL-6 is a particularly important driver of chemoresistance. Activation of the IL-6/JAK/STAT3 signaling axis enhances the expression of adenosine triphosphate (ATP)-binding cassette (ABC) transporters, including ATP-binding cassette subfamily B member 1 (ABCB1) and ATP-binding cassette subfamily G member 2 (ABCG2), while simultaneously promoting anti-apoptotic signaling and tumor cell survival^[46,47]^. HGF/cellular mesenchymal–epithelial transition factor (c-MET), TGF-β, and CXCL12-C-X-C motif chemokine receptor 4 (CXCR4) signaling further activate PI3K/PKB, MAPK, EMT, stemness, and cell adhesion programs, reducing chemotherapy sensitivity^[11,48,49]^.

### Immunosuppressive cellular network

PDAC is widely regarded as an immunologically “cold” tumor characterized by limited infiltration of cytotoxic immune cells and extensive accumulation of immunosuppressive populations^[22,50-52]^. The PDAC immune microenvironment is dominated by myeloid-derived suppressor cells (MDSCs), M2-polarized tumor-associated macrophages (TAMs), and regulatory T cells (Tregs), which establish a self-reinforcing immunosuppressive network^[53,54]^.

TAMs are key components of the PDAC resistance ecosystem because they integrate inflammatory, metabolic, and treatment-induced signals and translate these cues into tumor-supportive responses^[55]^. TAMs can promote chemoresistance through both soluble mediators and metabolic crosstalk. For example, macrophage-derived cytidine deaminase has been shown to reduce gemcitabine-induced apoptosis, whereas macrophage-released deoxycytidine can directly compete with gemcitabine for cellular uptake and metabolism, thereby decreasing its cytotoxic activity^[56]^. In addition, macrophages that engulf chemotherapy-induced apoptotic tumor cells can release 14-3-3ζ, activating AXL receptor tyrosine kinase (AXL)/PKB-dependent survival signaling in PDAC cells and promoting resistance to gemcitabine and nab-paclitaxel^[57]^. These observations indicate that chemotherapy can paradoxically reinforce a macrophage-dependent survival niche rather than simply eliminating tumor cells. TAMs may also contribute to immune checkpoint resistance by sustaining an immunosuppressive myeloid niche and limiting effective CD8^+^ T-cell infiltration and function. Importantly, TAM-targeted strategies may therefore need to focus on reprogramming macrophage states rather than indiscriminate depletion^[58,59]^.

Although immune suppression is traditionally associated with resistance to immunotherapy, recent mechanistic studies suggest that immunosuppressive cells also contribute significantly to chemotherapy resistance^[22,27,60]^. MDSCs produce arginase, inducible nitric oxide synthase, and reactive oxygen species, thereby depleting essential nutrients required for effective immune responses and simultaneously promoting tumor cell survival^[53,61]^. TAMs predominantly exhibit an M2 phenotype and secrete IL-10 and TGF-β, which enhance EMT, stemness, and resistance to apoptosis^[62]^. Moreover, Tregs suppress immune surveillance and facilitate the survival of resistant tumor clones during chemotherapy exposure. Together, these immunosuppressive populations create a protective niche that enables tumor cells to withstand cytotoxic treatment and promotes disease recurrence^[52]^.

### Hypoxia and tumor acidosis

Severe hypoxia is a hallmark of PDAC and results primarily from extensive stromal accumulation, vascular compression, and inadequate perfusion^[63-65]^. Persistent activation of hypoxia-inducible factor 1α (HIF-1α) induces a broad transcriptional program that supports tumor survival under therapeutic stress^[66]^. Hypoxia may alter the expression of drug-metabolizing enzymes and nucleotide-metabolism pathways, thereby modifying drug exposure and sensitivity. However, the relative contribution of these mechanisms in clinical PDAC remains incompletely defined^[67]^. In parallel, HIF-1α activates developmental pathways including Notch and Wnt signaling, leading to increased expression of stemness-associated transcription factors such as SRY-box transcription factor 2 (SOX2) and octamer-binding transcription factor 4 (OCT4)^[68,69]^. Cancer stem-like cells generated under hypoxic conditions exhibit enhanced DNA repair capacity, resistance to apoptosis, and reduced sensitivity to chemotherapy^[69]^. Moreover, hypoxia-induced VEGF expression promotes the formation of structurally abnormal blood vessels that further impair drug delivery and exacerbate therapeutic resistance^[45]^.

Hypoxia is closely coupled to extracellular acidosis in PDAC. Abnormal vascular perfusion, high glycolytic activity, and increased lactate production collectively contribute to the accumulation of protons and other acidic metabolites within the tumor interstitium. Acidosis can influence therapeutic response through both pharmacological and biological mechanisms. Low extracellular pH may impair the intracellular accumulation of weak-base chemotherapeutic agents through ion trapping and can induce adaptive programs involving drug efflux, autophagy, stress responses, and reduced apoptotic susceptibility^[70]^. In PDAC models, adaptation to an acidic extracellular environment has been shown to increase survival and reduce sensitivity to gemcitabine and erlotinib, providing direct evidence that acidosis can contribute to treatment resistance in pancreatic cancer^[71]^.

Tumor acidosis also interacts with immune and metabolic components of the TME. Acidic conditions can impair T-cell and natural killer-cell function while favoring a metabolically adapted tumor phenotype. Conversely, increased glycolytic activity and lactate production can further exacerbate extracellular acidification, generating a positive feedback loop between metabolic reprogramming, hypoxia, immune dysfunction, and therapeutic resistance^[72]^. Thus, hypoxia and acidosis should be considered interconnected physicochemical features of the PDAC resistance ecosystem rather than independent stress conditions.

### Metabolic reprogramming and metabolic-immune crosstalk

Metabolic reprogramming represents a hallmark of PDAC and is increasingly recognized as a major contributor to therapeutic resistance^[73-75]^. Unlike normal tissues, the PDAC microenvironment is characterized by severe nutrient deprivation, glucose scarcity, excessive lactate accumulation, amino acid imbalance, and dysregulated lipid metabolism. These metabolic alterations arise from the combined effects of rapid tumor growth, inadequate vascularization, and extensive stromal remodeling^[74,75]^. Importantly, tumor cells and stromal cells establish a metabolically cooperative ecosystem that supports survival under hostile conditions. One of the most extensively studied mechanisms is the reverse Warburg effect, in which CAFs undergo glycolytic reprogramming and produce large quantities of lactate. Lactate is subsequently exported through monocarboxylate transporter 4 (MCT4) and imported into PDAC cells via monocarboxylate transporter 1 (MCT1), where it fuels oxidative phosphorylation and ATP production^[74,76,77]^.

In addition to lactate metabolism, glutamine utilization plays a central role in PDAC progression. Tumor cells exhibit remarkable metabolic plasticity and exploit alternative glutamine metabolic pathways to sustain biosynthesis and redox homeostasis under nutrient-limited conditions^[75,78]^. Kirsten rat sarcoma viral oncogene homolog (KRAS)-driven glutamine utilization promotes nicotinamide adenine dinucleotide phosphate (NADPH) production and protects tumor cells from oxidative damage induced by chemotherapy. Disruption of glutamine metabolism has therefore emerged as a potential strategy for sensitizing PDAC cells to treatment^[74,75]^.

Furthermore, aberrant lipid metabolism supports membrane synthesis, energy storage, and antioxidant defense mechanisms, thereby enhancing resistance to chemotherapy and targeted therapy^[79,80]^. Increased fatty acid synthesis, lipid uptake, and β-oxidation provide both structural components for membrane biogenesis and alternative energy sources during metabolic stress. Lipid metabolic adaptation has also been linked to stemness maintenance, metastatic dissemination, and resistance to apoptosis. Several studies have demonstrated that inhibition of fatty acid metabolism can enhance chemosensitivity and suppress tumor progression in preclinical PDAC models^[77,78]^.

### EV-mediated intercellular communication

EVs are membrane-enclosed vesicles released by tumor and stromal cells that mediate intercellular communication within the PDAC microenvironment. EVs can transport biologically active cargo, including proteins, lipids, metabolites, and nucleic acids, to recipient cells and thereby modify their phenotypes^[81]^. In PDAC, such EV-mediated communication can transmit resistance-associated signals between malignant, stromal, and immune compartments of the TME^[82-85]^.

Importantly, macrophage-derived EVs represent a specific mechanism linking the immune microenvironment to chemoresistance. Macrophage-derived exosomes have been shown to transfer miR-365 to PDAC cells, increasing the intracellular nucleotide pool and cytidine deaminase expression and thereby reducing gemcitabine sensitivity^[82]^. Macrophage-derived EVs can also decrease PDAC sensitivity to gemcitabine; proteomic analyses identified chitinase 3-like 1 and fibronectin as abundant EV cargo proteins, and functional experiments linked these proteins to extracellular signal-regulated kinase (ERK)-dependent gemcitabine resistance^[83]^. These findings provide direct evidence that EV-mediated communication between immune cells and tumor cells can contribute to therapeutic resistance.

EVs also mediate bidirectional communication between tumor and stromal cells. CAF-derived EV-packaged circBIRC6 has been shown to promote platinum resistance in pancreatic cancer through modulation of SUMOylation^[84]^. Conversely, EV-packaged Linc-ZNF25-1 derived from pancreatic cancer cells promotes asparagine uptake by PSCs, enhancing PSC activation and ECM formation and thereby contributing to gemcitabine resistance^[85]^. Collectively, these findings indicate that EV-mediated signaling can promote therapeutic resistance through mechanisms determined by both the cellular origin of EVs and their specific molecular cargo. EVs may therefore represent potential biomarkers for treatment monitoring and resistance prediction, as well as potential therapeutic targets. Strategies to interfere with EV biogenesis, release, uptake, or defined resistance-associated cargo warrant further investigation. However, EV heterogeneity and methodological variability in isolation and characterization remain important barriers to clinical translation^[86]^.

### Neural and microbial components of the resistance ecosystem

The contribution of microbiota to PDAC biology has emerged as a rapidly evolving research field. Recent mechanistic studies have identified distinct intratumoral microbial communities within pancreatic tumors and demonstrated their involvement in tumor progression, immune regulation, and therapeutic resistance^[87,88]^. Certain bacterial species, particularly Gammaproteobacteria, express bacterial cytidine deaminase capable of metabolizing gemcitabine into inactive metabolites, thereby directly promoting chemoresistance^[87]^. Microbial dysbiosis may additionally modulate immune cell differentiation and alter responses to immunotherapy^[88,89]^.

Another emerging component of the PDAC microenvironment is the neuroimmune niche. Perineural invasion (PNI) is a pathological hallmark of PDAC and is strongly associated with disease progression and poor prognosis^[50]^. Neural fibers within the TME release neurotransmitters, including catecholamines and norepinephrine, which interact with adrenergic receptors expressed on macrophages, CAFs, and tumor cells^[50]^. This signaling facilitates M2 macrophage polarization, enhances secretion of IL-6 and VEGF, and facilitates establishment of an immunosuppressive niche^[50,90,91]^. As a result, neuroimmune interactions not only promote tumor invasion and metastasis but also contribute to resistance against chemotherapy, targeted therapy, and immune-mediated tumor elimination.

### Spatial heterogeneity

A growing body of evidence indicates that the PDAC microenvironment exhibits remarkable spatial heterogeneity, which plays a critical role in determining therapeutic response and disease progression^[17,26,92-94]^. Traditional bulk sequencing approaches have provided valuable molecular information; however, they fail to capture the spatial organization of distinct cellular populations within tumors^[95]^. Recent advances in single-cell RNA sequencing have revealed previously unrecognized heterogeneity among CAFs, immune cells, and malignant epithelial cells^[26,38]^. These studies have identified multiple CAF subpopulations, including myCAFs, iCAFs, apCAFs, and additional transitional states, highlighting the complexity of stromal regulation in PDAC^[26,37,38]^. The emergence of spatial transcriptomics and spatial proteomics has further transformed our understanding of PDAC biology. These technologies have demonstrated that CAF-rich regions frequently form physical barriers that exclude cytotoxic T cells from direct contact with tumor cells, thereby contributing to resistance to immune checkpoint inhibitors (ICIs)^[17]^. Likewise, hypoxic regions are often enriched with immunosuppressive macrophages, Tregs, and metabolically adapted tumor cells, creating specialized niches that promote therapeutic resistance^[17,63]^. Spatial multi-omics analyses have revealed that treatment-resistant tumors are characterized not only by specific molecular alterations but also by distinct spatial architectures involving stromal, immune, and metabolic compartments^[15,17]^.

## KEY HALLMARKS OF TME-MEDIATED THERAPEUTIC RESISTANCE

Beyond constitutive stromal, immune, hypoxic, and metabolic barriers, the PDAC microenvironment promotes adaptive cellular states that enable tumor persistence under therapeutic pressure. Five interconnected hallmarks are particularly relevant: cancer stem cell (CSC) plasticity, stress-adaptive autophagy, ferroptosis evasion, metabolic-immune crosstalk, and therapy-induced TME remodeling. These processes connect microenvironmental stress with tumor-cell adaptation and contribute to both intrinsic and acquired therapeutic resistance.

### CSC plasticity

CSCs represent a phenotypically plastic tumor-cell state characterized by self-renewal, tumor-initiating capacity, and enhanced tolerance to therapeutic stress^[96-98]^. In PDAC, CSC-enriched populations contribute to tumor propagation, metastatic dissemination, and treatment resistance. Importantly, stemness is not necessarily a fixed cellular identity but can be dynamically regulated by tumor-intrinsic programs and microenvironmental cues^[20,68,99]^.

The TME provides selective conditions that favor CSC persistence and plasticity. Hypoxia, nutrient limitation, stromal signaling, and inflammatory mediators can reinforce stem-like and epithelial-mesenchymal programs, generating protective niches in which residual tumor cells survive therapeutic stress. These interactions link microenvironmental adaptation to tumor repopulation and acquired resistance^[75,100]^.

Metabolic heterogeneity further contributes to CSC adaptation. Pancreatic CSCs are preferentially dependent on oxidative phosphorylation and display limited metabolic plasticity; however, metabolically flexible resistant clones can emerge under mitochondrial stress through alterations in the MYC proto-oncogene, bHLH transcription factor (MYC)/peroxisome proliferator-activated receptor gamma coactivator 1-alpha (PGC-1α) balance^[100]^. This adaptive metabolic heterogeneity may permit a subset of CSCs to survive metabolic or therapeutic perturbation and contribute to tumor recurrence.

### Stress adaptation and autophagy

Autophagy is a major stress-adaptive program that enables PDAC cells to survive hypoxia, nutrient deprivation, and therapeutic stress^[101,102]^. In hypoxic and nutrient-poor conditions, autophagic flux supports cellular homeostasis by recycling intracellular components and maintaining metabolic substrates. Notably, pancreatic tumor-initiating cells exhibit enhanced autophagy under hypoxic stress, linking microenvironmental deprivation to the survival of stem-like, therapy-resistant populations^[103]^.

Autophagy also operates within stromal compartments. PSCs use autophagy to release alanine, which can be taken up by PDAC cells and used to support central carbon metabolism under nutrient-limited conditions^[104]^. This metabolic exchange illustrates how stromal autophagy can support tumor-cell fitness within the nutrient-poor PDAC microenvironment.

Therapeutic stress can further induce protective autophagy. In Rat sarcoma viral oncogene homolog (RAS)-driven cancers, inhibition of the RAF family of serine/threonine protein kinases (RAF)-mitogen-activated protein kinase kinase (MEK)-ERK pathway induces autophagic flux that promotes tumor-cell survival, providing a mechanistic basis for combining MAPK-pathway inhibition with autophagy blockade^[105]^. Autophagy has also been associated with the maintenance of stem-like states, further linking stress adaptation with CSC persistence^[106]^.

Collectively, these findings identify autophagy as a context-dependent survival mechanism connecting microenvironmental stress with therapeutic adaptation. Although autophagy inhibition has shown preclinical activity, its clinical benefit in unselected PDAC remains unproven.

### Ferroptosis evasion

Ferroptosis is an iron-dependent form of regulated cell death driven by uncontrolled lipid peroxidation and failure of antioxidant defenses^[107,108]^. Suppression of ferroptotic stress represents an important survival mechanism in PDAC, in which oncogenic and metabolic programs generate substantial oxidative stress.

PDAC exhibits a marked dependence on extracellular cysteine for ferroptosis suppression. Cystine import through system Xc^-^, mediated by the SLC7A11/SLC3A2 transporter complex, supplies cysteine for the synthesis of glutathione and coenzyme A, thereby limiting lipid peroxidation. Genetic deletion of SLC7A11 or systemic cystine depletion induced tumor-selective ferroptosis and suppressed PDAC growth in genetically engineered mouse models^[109]^.

The relationship between ferroptosis and the PDAC microenvironment is bidirectional. Microenvironmental conditions can influence nutrient availability, redox balance, and lipid metabolism, thereby altering ferroptosis sensitivity; conversely, ferroptotic damage can remodel inflammatory signaling within pancreatic tumors^[110-112]^. Thus, ferroptosis should be considered within the broader metabolic and inflammatory context of the PDAC resistance ecosystem rather than as an exclusively tumor-cell-intrinsic process.

These dependencies provide a rationale for ferroptosis-sensitizing strategies; however, current evidence in PDAC remains predominantly preclinical, and the therapeutic window and context dependence of ferroptosis induction require further investigation.

### Metabolic-immune crosstalk

Metabolic reprogramming in PDAC affects therapeutic response not only by sustaining tumor-cell survival but also by reshaping antitumor immunity. Competition for nutrients and accumulation of immunomodulatory metabolites create reciprocal interactions among malignant, stromal, and immune cells, linking metabolic adaptation to immune dysfunction and therapeutic resistance. Lactate and adenosine provide particularly well-defined examples of this metabolic-immune crosstalk in PDAC^[23,113]^.

Lactate provides a direct mechanistic link between tumor metabolism and immune suppression. Recent PDAC-specific evidence shows that increased glycolytic lactate promotes K63 lactylation of endosulfine-α (ENSA), sustaining SRC-STAT3 signaling and C-C motif chemokine ligand 2 (CCL2) production in tumor cells. CCL2 promotes TAM recruitment, while lactate also reprograms macrophages toward an immunosuppressive state. Disrupting lactate production or the ENSA-STAT3-CCL2 axis restored sensitivity to immune checkpoint blockade in preclinical PDAC models^[114]^.

The CD73-adenosine axis represents another PDAC-specific metabolic-immune pathway. CD73-mediated adenosine production promotes immunosuppression through both myeloid and lymphoid compartments. In PDAC models, CD73 has been linked to granulocyte-macrophage colony-stimulating factor (GM-CSF)-dependent accumulation of MDSCs and impaired T-cell activity, while tumor-cell-autonomous adenosine receptor signaling can activate p38-STAT1-CCL5 signaling and promote regulatory T-cell recruitment^[115,116]^.

Additional metabolic processes, including tryptophan catabolism and competition for glutamine and other nutrients, may further constrain antitumor immunity, although their direct contribution to therapeutic resistance in PDAC is less well defined^[78,79,117]^. Collectively, these findings demonstrate that metabolic adaptation and immune suppression are mechanistically coupled rather than parallel features of the PDAC TME. Lactate-driven macrophage remodeling and CD73-dependent adenosine signaling provide direct examples in which metabolic alterations generate immunosuppressive cellular states and reduce sensitivity to antitumor immunity, supporting mechanism-guided metabolic-immunotherapeutic combinations.

### Therapy-induced evolution of the TME

The PDAC resistance ecosystem is dynamically remodeled by treatment. Chemotherapy, radiotherapy, targeted therapy, and immunotherapy not only select resistant tumor-cell states but can also alter stromal composition, immune-cell recruitment, metabolic dependencies, and intercellular signaling. These treatment-induced changes generate new selective pressures and protective niches that support residual disease and acquired resistance^[18,47,118,119]^.

KRAS-pathway inhibition provides a representative example of therapy-induced ecosystem adaptation. Although KRAS inhibition suppresses tumor-cell signaling, residual PDAC cells can engage compensatory MAPK and bypass pathways, while the surrounding stroma undergoes functional remodeling that can limit durable response. Preclinical studies further indicate that stromal reprogramming can restore sensitivity to RAS-MAPK inhibition and enhance responses to cytotoxic and immune therapies^[120]^.

These observations support a co-evolutionary model of acquired resistance in which therapy simultaneously selects tumor-cell states and reshapes the surrounding microenvironment. CSC plasticity, autophagy, ferroptosis evasion, metabolic-immune crosstalk, and stromal or immune remodeling should therefore not be viewed as isolated mechanisms, but as interconnected adaptive responses that emerge under therapeutic pressure. This dynamic perspective provides the rationale for longitudinal biomarker assessment and mechanism-guided combination therapy rather than sequential targeting of individual resistance pathways.

## TME-MEDIATED RESISTANCE ACROSS THERAPEUTIC MODALITIES

The PDAC TME limits therapeutic efficacy through mechanisms that are shared across treatment modalities but differ in their relative contribution to each therapeutic context. Physical drug exclusion, stromal signaling, hypoxia, metabolic adaptation, immune suppression, and therapy-induced plasticity collectively shape responses to chemotherapy, targeted therapy, immunotherapy, and radiotherapy. Figure 2 summarizes the multilevel mechanisms of therapeutic resistance in PDAC driven by the TME.

**Figure 2:**
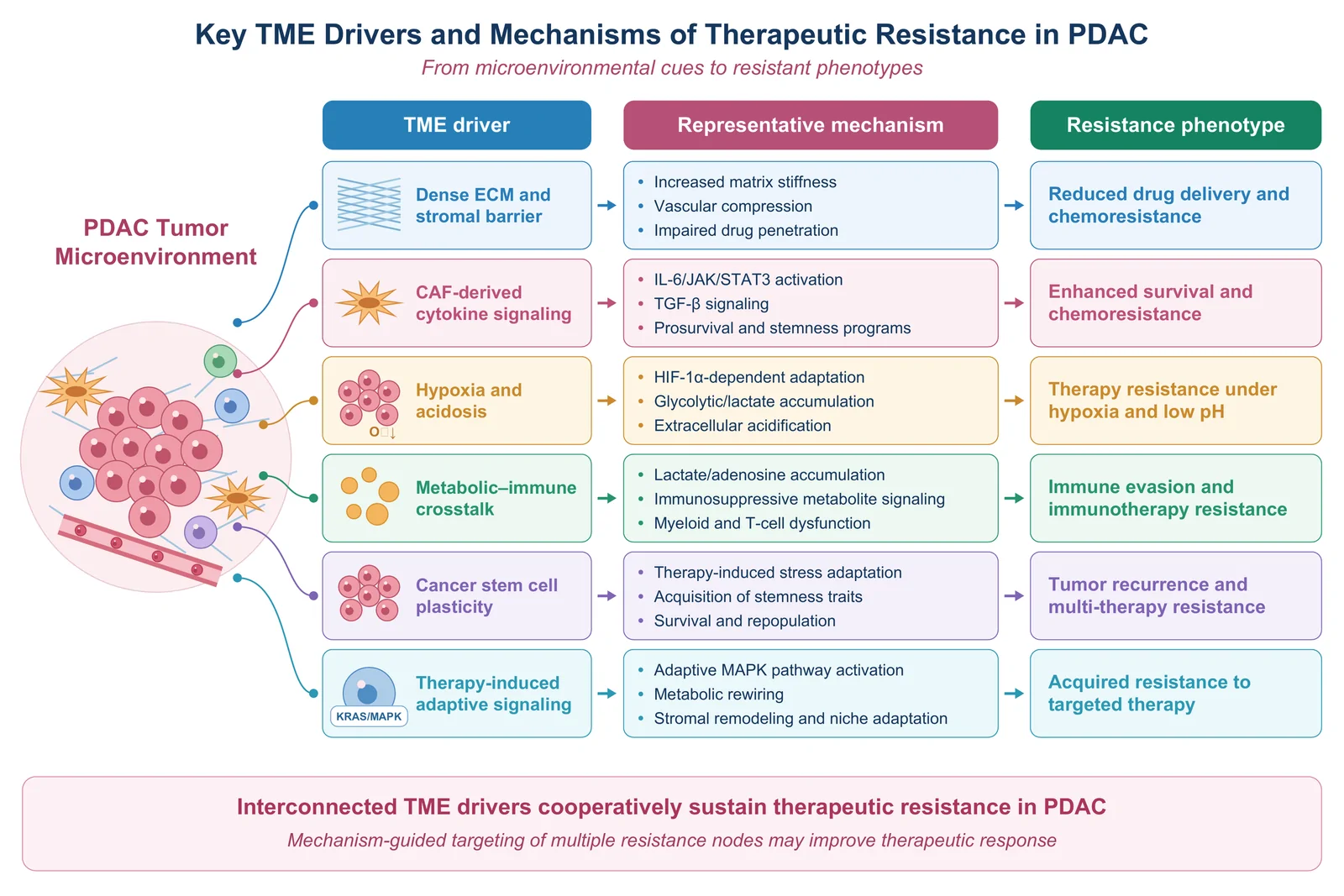
Key TME drivers and mechanisms of therapeutic resistance in PDAC. Major components and stressors within the PDAC TME promote distinct but interconnected resistance phenotypes. Dense ECM deposition and stromal remodeling increase matrix stiffness, compress tumor vasculature, and impair drug penetration, thereby limiting chemotherapy delivery. CAF-derived cytokine signaling activates prosurvival and stemness-associated pathways, including IL-6/JAK/STAT3 and TGF-β signaling, which contribute to chemoresistance. Hypoxia and extracellular acidosis promote HIF-1α-dependent adaptation, glycolytic/lactate accumulation, and survival under low-pH conditions. Metabolic–immune crosstalk involving lactate, adenosine, and other immunosuppressive metabolites contributes to myeloid and T-cell dysfunction and immune escape. Therapy-induced stress can also promote CSC plasticity, survival, and tumor repopulation, whereas adaptive MAPK signaling, metabolic rewiring, and stromal remodeling can facilitate acquired resistance to targeted therapy. Collectively, these interconnected mechanisms illustrate how multiple TME-derived resistance nodes cooperate to sustain therapeutic resistance in PDAC and provide a rationale for mechanism-guided combination strategies. TME: Tumor microenvironment; PDAC: pancreatic ductal adenocarcinoma; ECM: extracellular matrix; CAF: cancer-associated fibroblast; IL-6: interleukin-6; JAK: Janus kinase; STAT3: signal transducer and activator of transcription 3; TGF-β: transforming growth factor-β; HIF-1α: hypoxia-inducible factor 1α; CSC: cancer stem cell; MAPK: mitogen-activated protein kinase.

### Chemotherapy

Chemotherapy efficacy in PDAC is constrained by impaired drug delivery and microenvironment-mediated survival signaling. Dense deposition of collagen and hyaluronan increases interstitial pressure and compresses intratumoral vessels, reducing perfusion and drug penetration^[9,28,32,33]^. In parallel, ECM-integrin interactions activate FAK, PI3K-PKB, and MAPK signaling, linking stromal mechanics to prosurvival signaling and reduced chemotherapy sensitivity^[35]^.

CAFs and PSCs further promote chemoresistance through paracrine signaling and extracellular communication. CAF-derived IL-6 activates JAK-STAT3 signaling, whereas HGF-c-MET, TGF-β, and CXCL12-CXCR4 signaling reinforce prosurvival, epithelial-mesenchymal, and stem-like programs^[11,12,45-49]^. EVs provide an additional route of stromal-tumor communication by transferring proteins and non-coding RNAs that alter survival signaling and drug sensitivity^[66,121-123]^. Together, these mechanisms enable stromal cells to regulate chemotherapy response beyond their role as a physical barrier.

Myeloid cells can also directly alter chemotherapy efficacy. In PDAC, tumor-conditioned macrophages release pyrimidine nucleosides, particularly deoxycytidine, which competes with gemcitabine for cellular uptake and metabolism, thereby reducing its cytotoxic activity. Macrophage depletion enhanced gemcitabine responsiveness in preclinical PDAC models, providing direct evidence that TAM metabolism can determine chemotherapy sensitivity^[56]^.

Hypoxia and metabolic stress further reinforce chemoresistance by promoting stem-like states, redox adaptation, and stress-survival programs, including autophagy^[63-65,68,69,101,102]^. These adaptive processes converge with impaired drug delivery and stromal signaling to permit the persistence of residual tumor cells during chemotherapy. Thus, chemoresistance in PDAC reflects coordinated physical, stromal, metabolic, and cellular mechanisms rather than a single drug-specific pathway.

### Targeted therapy

Resistance to targeted therapy in PDAC arises from reciprocal interactions between tumor-intrinsic signaling and the surrounding microenvironment. Stromal signaling, ECM-mediated mechanotransduction, hypoxia, and metabolic adaptation can provide compensatory survival signals that reduce tumor-cell dependence on the therapeutically inhibited pathway. Targeted-therapy resistance therefore cannot be explained solely by secondary tumor-cell alterations.

KRAS inhibition provides a representative example of this reciprocal interaction. In KRAS G12D-mutant PDAC models, extracellular context and integrin β1 (ITGB1)-dependent mechanotransduction regulate sensitivity to MRTX1133 through yes-associated protein (YAP)/transcriptional coactivator with PDZ-binding motif (TAZ) signaling. Moreover, KRAS G12D inhibition remodeled the immune microenvironment, with tumor regression in immunocompetent models associated with reduced neutrophil abundance and increased effector CD8^+^ T-cell infiltration^[124]^. These findings demonstrate that both tumor-intrinsic signaling and the extracellular and immune microenvironment determine the response to KRAS inhibition.

Therapeutic pressure can further induce compensatory MAPK signaling, metabolic rewiring, and stromal remodeling, enabling residual tumor cells to recover fitness after pathway inhibition^[120]^. These observations support a co-evolutionary model in which KRAS-targeted therapy reshapes both tumor-cell signaling and the TME, providing a rationale for combinations that simultaneously suppress oncogenic signaling and microenvironmental escape mechanisms.

### Immunotherapy

PDAC is characterized by a profoundly immune-excluded TME that limits the efficacy of immune checkpoint blockade. Dense ECM and CAF-rich stromal regions restrict cytotoxic T-cell infiltration, whereas hypoxia and metabolic stress further impair T-cell survival and effector function^[22,125-127]^. Thus, resistance frequently arises upstream of checkpoint engagement because functional effector T cells are scarce within tumor-cell-rich regions.

Myeloid and regulatory lymphoid populations reinforce this immune-excluded state. TAMs suppress T-cell recruitment and effector function through immunosuppressive cytokines and inhibitory signaling, whereas MDSCs impair T-cell activation through arginase-1-mediated L-arginine depletion and reactive oxygen and nitrogen species. Tregs further constrain antitumor immunity through IL-10 and TGF-β and by disrupting productive interactions between dendritic cells and cytotoxic CD8^+^ T cells^[128,129]^.

Metabolic immune suppression further compounds cellular immune exclusion. Lactate accumulation and adenosine signaling impair effector lymphocyte function, while defective dendritic-cell activation and antigen presentation limit effective tumor recognition^[23,113]^. These overlapping physical, cellular, and metabolic barriers help explain why blockade of a single immune checkpoint is generally insufficient in unselected PDAC and support strategies that combine checkpoint inhibition with TME remodeling.

### Radiotherapy

Radiotherapy efficacy in PDAC is strongly constrained by hypoxia. Dense desmoplasia and vascular compression reduce tumor perfusion and oxygenation, whereas oxygen deficiency limits fixation of radiation-induced DNA damage and thereby reduces radiosensitivity^[130-133]^. Hypoxia-inducible signaling additionally promotes antioxidant defense, metabolic adaptation, and survival programs that increase tolerance to radiation-induced stress.

Radiotherapy also interacts dynamically with the stromal and immune microenvironment. In PDAC models, fibroblasts and collagen cooperate to impair radiation efficacy and radiation-induced interferon signaling. FAK inhibition reprogrammed the stroma, restored radiation-induced T-cell priming, and enhanced responses to checkpoint blockade, demonstrating that stromal signaling can directly determine radiotherapy response^[134]^.

Radiotherapy can therefore exert opposing effects on the PDAC ecosystem: tumor-cell damage may promote antigen release and immune priming, whereas persistent hypoxia, stromal fibrosis, and myeloid-mediated immunosuppression can constrain these responses. Radiosensitivity consequently depends not only on tumor-cell DNA damage but also on the state of the surrounding TME, providing a rationale for combining radiotherapy with mechanism-based stromal or immune modulation.

## THERAPEUTIC TARGETING OF THE PDAC RESISTANCE ECOSYSTEM

Given the central role of the resistance ecosystem in PDAC progression and therapeutic resistance, considerable efforts have been devoted to developing strategies that target stromal remodeling, hypoxia, immune suppression, and metabolic adaptation. Increasing evidence suggests that effective treatment of PDAC requires not only direct elimination of malignant cells but also simultaneous modulation of the supportive microenvironment^[14,29,135]^. A comprehensive overview of current and emerging therapeutic strategies targeting distinct components of the PDAC resistance ecosystem, including TME-directed and tumor-intrinsic/TME-interacting approaches, is presented in Table 1.

**Table 1:** Current and emerging therapeutic strategies targeting the PDAC resistance ecosystem

**Therapeutic compartment**	**Target/strategy**	**Representative intervention**	**Evidence level**	**Mechanistic rationale**	**Clinical evidence and major limitation**	**Ref.**
Stromal/ECM	Hyaluronan depletion	PEGPH20 (pegvorhyaluronidase alfa) + gemcitabine/nab-paclitaxel	Randomized clinical	Degrades hyaluronan and reduces ECM-associated physical constraints on tumor perfusion and drug distribution	HALO-301 (Phase III): ORR increased from 36% to 47%, but median OS (11.2 *vs.* 11.5 months) and PFS (7.1 *vs.* 7.1 months) were not improved. These findings challenge indiscriminate stromal depletion as a therapeutic strategy	[136]
Stromal/CAF-PSC	Stromal reprogramming	Paricalcitol; other VDR agonists	Early clinical	Promotes a less activated stromal phenotype and may modify fibroblast composition and immune accessibility without eliminating the stroma	A randomized run-in study (NCT03520790) showed modulation of fibroblast and CD8^+^ T-cell features, but was not designed to establish survival benefit. Another phase II study of oral paricalcitol (NCT04617067) was stopped early for failure to meet its prespecified efficacy criterion	[137,138]
Stromal/CAF	FAK inhibition and stromal reprogramming	Defactinib (VS-6063) + SBRT	Early clinical/preclinical	FAK inhibition can counteract fibroblast- and collagen-mediated resistance, restore radiation-induced interferon signaling, and promote T-cell priming	NCT04331041: clinical evaluation of defactinib with SBRT was supported by preclinical and tissue-level evidence of stromal reprogramming; definitive clinical efficacy remains to be established	[134]
Immune/myeloid	CCL2-CCR2 axis	PF-04136309 + chemotherapy	Early clinical	CCR2 blockade reduces recruitment of inflammatory monocytes and TAMs, thereby attenuating myeloid-mediated immunosuppression	Phase Ib studies evaluated PF-04136309 with FOLFIRINOX or gemcitabine/nab-paclitaxel. Although biological activity was observed, subsequent development has not established a definitive clinical benefit	[139,140]
Immune/stromal	CXCL12-CXCR4 axis	Plerixafor + cemiplimab	Early clinical	CXCR4 blockade facilitates T-cell recruitment into the tumor and is intended to enhance the activity of PD-1 blockade	Phase II: among 21 patients with metastatic PDAC, only two achieved stable disease as their best response. Increased immune infiltration was accompanied by enrichment of immunosuppressive macrophages, illustrating compensatory resistance within the TME	[141]
Immune/adenosine	CD73-adenosine axis	Oleclumab ± durvalumab + chemotherapy	Randomized phase Ib/II	CD73 inhibition reduces adenosine-mediated immunosuppression and may increase immune responsiveness to checkpoint blockade	NCT03611556: the randomized phase II comparison did not meet its primary efficacy endpoint; ORR were 29.0%, 21.1%, and 32.9% across the three treatment arms, with no significant PFS or OS improvement for the combination arm	[142]
Immune/myeloid-stromal	CD40 activation	Agonistic CD40 antibody + chemotherapy	Preclinical/early clinical	CD40 activation promotes macrophage-mediated tumor killing and can remodel tumor stroma, providing a mechanism for combining immune activation with chemotherapy	Preclinical models and an early clinical study demonstrated biological and antitumor activity, but CD40-based strategies remain investigational and require validation in larger controlled studies	[143]
Tumor-intrinsic/TME-interacting	KRAS signaling	Sotorasib (KRAS G12C); MRTX1133 (KRAS G12D)	Early clinical (KRAS G12C inhibition); predominantly preclinical/developmental (KRAS G12D inhibition)	Suppresses oncogenic KRAS signaling; combination with TME-directed approaches may limit adaptive stromal and signaling responses	Sotorasib has demonstrated clinical activity in KRAS G12C-mutant advanced PDAC, whereas KRAS G12D-directed strategies remain less clinically established. Adaptive MAPK signaling, stromal remodeling, and metabolic compensation may limit durable responses	[144,145]
Integrated tumor-stroma signaling	FAK-RAS/MAPK crosstalk	FAK inhibitor + RAF-MEK inhibitor	Preclinical	CAF-mediated signaling can sustain MYC and adaptive resistance during RAF-MEK inhibition; combined FAK and RAF-MEK inhibition therefore targets complementary tumor-stroma resistance mechanisms	Combined inhibition produced tumor growth suppression and improved survival across multiple PDAC mouse models, providing a mechanistic rationale for future combination development; clinical efficacy has not yet been established	[120]

Evidence levels reflect the highest level of PDAC-specific evidence supporting each strategy and should not be interpreted as evidence of clinical efficacy. Clinical-trial evaluation indicates investigation rather than established therapeutic benefit. PDAC: Pancreatic ductal adenocarcinoma; ECM: extracellular matrix; PEGPH20: pegylated recombinant human hyaluronidase; ORR: objective response rate; OS: overall survival; PFS: progression-free survival; CAF: cancer-associated fibroblast; PSC: pancreatic stellate cell; VDR: vitamin D receptor; FAK: focal adhesion kinase; SBRT: stereotactic body radiotherapy; CCL2: C-C motif chemokine ligand 2; CCR2: C-C motif chemokine receptor 2; TAMs: tumor-associated macrophages; CXCL12: C-X-C motif chemokine ligand 12; CXCR4: C-X-C motif chemokine receptor 4; PD-1: programmed cell death protein 1; TME: tumor microenvironment; KRAS: Kirsten rat sarcoma viral oncogene homolog; MAPK: mitogen-activated protein kinase; RAS: Rat sarcoma viral oncogene homolog; RAF: RAF family of serine/threonine protein kinases; MEK: mitogen-activated protein kinase kinase; MYC: MYC proto-oncogene, bHLH transcription factor.

### Stromal reprogramming and signaling modulation

Therapeutic targeting of the PDAC stroma has shifted from nonspecific stromal depletion toward selective remodeling and reprogramming. Early approaches focused on degrading hyaluronan (HA) to reduce ECM density, interstitial pressure, and barriers to drug delivery^[146]^. However, the failure of PEGPH20 to improve survival in the phase III HALO-301 trial showed that matrix depletion alone is insufficient to overcome PDAC therapeutic resistance^[136]^. The stroma is not merely a physical barrier but a dynamic compartment that regulates vascular function, immune responses, tumor-cell signaling, and treatment adaptation.

Preclinical studies support stromal reprogramming as an alternative to stromal depletion. Activation of the vitamin D receptor (VDR) in PSCs with calcipotriol restored a more quiescent stromal phenotype, reduced fibrosis and inflammation, increased intratumoral gemcitabine accumulation, and improved the response to chemotherapy in PDAC models^[147]^. These findings indicate that modifying stromal function may enhance treatment efficacy while preserving the structural functions of the tumor stroma.

This concept is further supported by the functional heterogeneity of CAFs. In genetically engineered PDAC models, depletion of α-SMA-positive myofibroblasts increased tumor aggressiveness, immunosuppression, and disease progression, whereas other studies showed that stromal elements can restrain tumor growth^[148,149]^. Thus, indiscriminate CAF depletion may disrupt tumor-restraining functions, supporting selective targeting of tumor-promoting stromal states rather than global stromal elimination.

Beyond stromal abundance and cellular state, therapeutic resistance can be driven by stromal signaling. TGF-β, HGF/c-MET, and IL-6/JAK/STAT3 are among the pathways through which stromal cells communicate with tumor cells and regulate survival, inflammation, and treatment sensitivity. TGF-β signaling contributes to fibrosis and immune suppression, whereas stromal-derived HGF can activate c-MET-dependent survival pathways in PDAC cells^[150,151]^. These overlapping signaling networks may allow tumor cells to compensate for inhibition of individual therapeutic targets.

The clinical experience with TGF-β inhibition also illustrates the gap between mechanistic rationale and therapeutic efficacy. In a phase Ib study, the TGF-β receptor I inhibitor galunisertib was combined with the anti-PD-L1 antibody durvalumab in patients with metastatic pancreatic cancer^[152]^. The study supported the feasibility of combined TGF-β and immune checkpoint targeting but provided limited evidence of durable clinical benefit, emphasizing that inhibition of a single stromal pathway may be insufficient when parallel resistance mechanisms remain active.

FAK signaling provides a further example of stromal adaptation to therapeutic pressure. In PDAC models, FAK inhibition reprogrammed the fibrotic stroma, restored radiation-induced interferon signaling, and enhanced T-cell priming and response to radiotherapy^[134]^. More recently, FAK inhibition was shown to overcome resistance to RAS-MAPK inhibition by altering CAF-mediated signaling; CAFs maintained MYC expression and produced fibroblast-derived factors such as FGF1 that contributed to resistance^[120]^. Combined FAK and RAF-MEK inhibition improved tumor control and survival in multiple PDAC models. These findings support a shift from targeting individual stromal components toward disrupting the adaptive interactions between stromal and tumor cells.

### Remodeling the immune microenvironment

Restoration of antitumor immunity is a major objective of current PDAC therapeutic development. However, successful immunotherapy requires reversal of the profound immune suppression present within the PDAC microenvironment^[22,125]^. Several approaches have been investigated to eliminate or reprogram immunosuppressive populations. Strategies targeting MDSCs, TAMs, and Tregs have demonstrated the potential to restore T-cell activity and enhance immune surveillance^[153-155]^. Particular attention has recently focused on macrophage reprogramming. Converting M2-like TAMs into tumoricidal M1 macrophages may simultaneously reduce immune suppression and improve responsiveness to ICIs^[153]^. In addition, inhibition of the CD39-CD73-adenosine axis has emerged as a highly promising strategy^[156]^. Extracellular adenosine suppresses T-cell activation through A2A and A2B receptors, thereby contributing to immune escape. Several anti-CD39 and anti-CD73 agents, including IPH5201 and oleclumab, are currently being evaluated in combination with PD-1 inhibitors and chemotherapy^[156]^. However, the magnitude of benefit remains modest, suggesting that adenosine blockade alone is unlikely to fully reverse immune exclusion in PDAC. The ultimate goal of these approaches is to convert immunologically “cold” PDAC tumors into “hot” tumors capable of mounting effective antitumor immune responses^[17]^.

### Hypoxia and metabolic targeting

Hypoxia and metabolic adaptation are closely interconnected features of the PDAC microenvironment. Rather than treating them as independent resistance mechanisms, increasing evidence indicates that oxygen limitation drives metabolic plasticity, including enhanced glycolysis, altered glutamine and lipid utilization, and maintenance of redox homeostasis, thereby supporting tumor-cell survival and therapy resistance^[157]^. HIF signaling is a central regulator of these adaptations and also contributes to an immunosuppressive microenvironment^[65]^. Therefore, therapeutic strategies targeting this axis may focus on disrupting hypoxia adaptation or selectively exploiting the metabolic dependencies that arise under hypoxic conditions.

Several preclinical studies support this approach. In pancreatic cancer models, inhibition of HIF-1α with PX-478 enhanced radiosensitivity, including when combined with gemcitabine or 5-fluorouracil, indicating that interference with hypoxia adaptation can increase the efficacy of cytotoxic treatment^[158]^. Hypoxia-activated prodrugs represent an alternative strategy that exploits the spatial heterogeneity of oxygen availability. However, in a randomized phase II trial, addition of the hypoxia-activated prodrug TH-302 to gemcitabine improved progression-free survival but did not establish a significant overall-survival benefit, highlighting the limitations of targeting hypoxia without appropriate patient selection or combination strategies^[159]^.

Metabolic interventions should similarly be considered within the context of TME adaptation rather than as isolated metabolic targets. Hypoxia-induced glycolysis and lactate accumulation can alter extracellular acidity and nutrient availability, while metabolic exchange between cancer cells and stromal cells may provide alternative substrates that sustain tumor growth and treatment resistance^[65,160]^. Consequently, simultaneous disruption of hypoxia adaptation and compensatory metabolic pathways may be more rational than inhibiting a single metabolic process. Importantly, these approaches remain predominantly preclinical, and the clinical benefit of metabolism-directed therapy in PDAC remains to be established.

Overall, current evidence supports a shift from broadly targeting “hypoxic” or “metabolically active” tumors toward biomarker-guided intervention of specific hypoxia-metabolism dependencies^[157,161]^. Because the degree and spatial distribution of hypoxia vary among PDAC tumors, hypoxia signatures or other pharmacodynamic biomarkers may be important for patient selection and for identifying tumors most likely to benefit from hypoxia- or metabolism-directed combinations.

### Emerging combination therapeutic strategies

Because therapeutic resistance in PDAC arises from interactions between tumor-intrinsic signaling and the TME, emerging strategies increasingly combine tumor-directed therapies with stromal or immune modulation. For example, combined FAK and RAF-MEK inhibition overcame CAF-mediated resistance to MAPK inhibition and enhanced responses to chemotherapy and immunotherapy in preclinical PDAC models, supporting a multi-pathway rather than single-target approach^[120]^.

Stromal reprogramming may also improve the efficacy of radiotherapy and immunotherapy. FAK inhibition restored radiation-induced interferon signaling, promoted T-cell priming, and enhanced responses to checkpoint blockade in PDAC models, providing the rationale for clinical evaluation of defactinib with stereotactic body radiotherapy (SBRT, NCT04331041)^[134]^. These findings support functional stromal reprogramming rather than indiscriminate stromal depletion.

Targeting immune-stromal interactions provides another potential strategy. Although CXCR4 blockade increased immune-cell infiltration in a phase II study of metastatic PDAC, clinical activity was limited and treatment was accompanied by enrichment of immunosuppressive macrophages, highlighting the compensatory mechanisms that may constrain TME-targeted therapy^[141]^. CD40 agonism represents an alternative approach, activating tumoricidal macrophages and remodeling tumor stroma; early clinical and preclinical studies provided evidence supporting its combination with chemotherapy^[143]^.

Overall, current evidence supports mechanism-guided combination therapy targeting complementary tumor, stromal, and immune vulnerabilities. However, most strategies remain supported by preclinical or early-phase evidence, emphasizing the need for biomarker-guided patient selection and rigorous clinical validation.

## CLINICAL TRANSLATION OF TME-TARGETED THERAPEUTIC STRATEGIES

### Preclinical evidence: TME targeting as a strategy to reverse therapeutic resistance

Most TME-directed strategies in PDAC remain supported primarily by preclinical evidence derived from genetically engineered mouse models, patient-derived xenografts, organoids, co-culture systems, and *ex vivo* human tumor models^[24,162]^. These studies have provided mechanistic evidence that stromal, immune, vascular, metabolic, and extracellular components of the TME can actively regulate therapeutic response rather than merely reflect tumor progression. Accordingly, TME-targeted approaches have focused on stromal remodeling, reprogramming of immunosuppressive cell populations, normalization of abnormal vasculature, modulation of hypoxia and metabolism, and interference with intercellular signaling.

Importantly, the preclinical literature increasingly supports TME reprogramming rather than indiscriminate stromal depletion. For example, inhibition of FAK has been shown to remodel the fibrotic stroma and enhance the response of PDAC to radiotherapy. In preclinical models, FAK inhibition restored radiation-induced interferon signaling, promoted tumor-specific T-cell priming, and enhanced antitumor responses when combined with immune checkpoint blockade^[134]^. These findings provided the mechanistic rationale for subsequent clinical investigation of FAK inhibition in combination with SBRT.

More recently, preclinical evidence has further demonstrated that stromal reprogramming can influence resistance to RAS/MAPK pathway inhibition. Long-term FAK inhibition was found to induce hyperactivation of the RAS/MAPK pathway in PDAC models and in post-treatment human PDAC tissues. Concomitant inhibition of FAK and RAF-MEK signaling produced greater tumor growth inhibition and prolonged survival in multiple PDAC mouse models. Mechanistically, CAFs impaired the downregulation of MYC following RAF-MEK inhibition, whereas FAK inhibition reprogrammed CAFs and reduced the production of fibroblast-derived growth factors, including FGF1, that contributed to resistance^[120]^.

These findings illustrate an important principle of the PDAC resistance ecosystem: the therapeutic relevance of the TME may arise not only from the presence of individual stromal or immune components, but also from their ability to dynamically compensate for therapeutic perturbations imposed on tumor cells. However, these observations remain predominantly preclinical and should not be interpreted as evidence of clinical efficacy.

### Early clinical evidence: translating stromal reprogramming into combination therapy

Several TME-directed approaches have progressed into early-phase clinical investigation, although clinical evidence remains considerably more limited than the extensive preclinical literature. In this setting, FAK inhibition represents one of the more mechanistically developed examples because its clinical evaluation has been directly informed by experimental studies of stromal-mediated therapeutic resistance^[134]^.

The RAMP205 study (NCT05669482) is a Phase 1b/2 study evaluating the combination of the RAF-MEK clamp inhibitor avutometinib and the FAK inhibitor defactinib with gemcitabine plus nab-paclitaxel in previously untreated patients with metastatic PDAC. The trial was designed to evaluate the safety, tolerability, and preliminary efficacy of simultaneously targeting MAPK signaling and FAK-dependent stromal signaling in combination with standard chemotherapy. ClinicalTrials.gov currently lists the study as Active, not recruiting^[163,164]^.

FAK inhibition has also been investigated as a strategy to improve radiation response. The NCT04331041 study evaluated defactinib in combination with SBRT in patients with advanced pancreatic adenocarcinoma following standard chemotherapy. This Phase II clinical investigation was motivated by preclinical evidence showing that FAK inhibition could reprogram the fibrotic tumor stroma, restore radiation-induced immune signaling, and facilitate T-cell priming. In the corresponding translational study, analysis of paired patient samples demonstrated stromal changes following treatment, providing clinical biological evidence consistent with the proposed mechanism^[134,165]^.

These studies illustrate a potentially important direction for TME-targeted therapy: rather than attempting to eliminate the entire stromal compartment, therapeutic intervention can be designed to modify the functional state of stromal cells and their interactions with tumor cells and immune cells. Nevertheless, these approaches remain under clinical evaluation, and mechanistic evidence or early clinical feasibility should not be equated with demonstrated survival benefit.

### Randomized clinical evidence: lessons from stromal depletion

The clinical development of PEGPH20 illustrates the limitations of translating a biologically plausible TME-targeting strategy directly into clinical benefit. HA accumulation is a prominent feature of the PDAC ECM and has been associated with elevated interstitial pressure and impaired tissue perfusion. PEGPH20 was developed to enzymatically degrade HA and thereby remodel the ECM and potentially improve drug delivery^[146,166]^.

Early clinical studies generated encouraging signals, particularly in patients with HA-high tumors. In the randomized Phase II HALO-202 study, PEGPH20 combined with nab-paclitaxel and gemcitabine produced a greater progression-free survival benefit in the HA-high subgroup, supporting the hypothesis that stromal composition might be therapeutically exploitable^[167]^.

However, this promising signal was not confirmed in the subsequent randomized Phase III HALO-301 trial. Among patients with previously untreated, metastatic, HA-high PDAC, PEGPH20 plus nab-paclitaxel/gemcitabine increased the objective response rate compared with placebo plus chemotherapy (47% *vs.* 36%) but failed to improve either overall survival [11.2 *vs.* 11.5 months; hazard ratio (HR), 1.00; *P* = 0.97] or progression-free survival (7.1 *vs.* 7.1 months; HR, 0.97). Consequently, the study did not support further development of PEGPH20 in metastatic PDAC^[136]^.

The HALO-301 trial illustrates the limitations of a simplistic “more stroma is worse, therefore stroma should be depleted” model. Although HA depletion can modify the physical properties of the tumor stroma and increase drug delivery under certain experimental conditions, depletion of a single ECM component does not necessarily result in normalization of the entire TME. The biological consequences of stromal manipulation may depend on the cellular composition, spatial organization, mechanical properties, vascular function, and compensatory signaling networks within the tumor.

This distinction is particularly important for the interpretation of TME-targeted therapies in PDAC. The failure of PEGPH20 suggests that therapeutic strategies directed against a single structural component may be insufficient when multiple interconnected mechanisms maintain treatment resistance. Consequently, future approaches may need to focus on context-dependent stromal reprogramming and rational combination therapy, guided by biomarkers that identify tumors in which a particular TME dependency is functionally relevant.

### Evidence-based perspective on TME-targeted therapy

Taken together, current evidence supports a cautious interpretation of TME-targeted therapy in PDAC. Preclinical studies have established multiple mechanisms through which CAFs, ECM, immune cells, vascular abnormalities, hypoxia, metabolic adaptations, and intercellular communication can contribute to therapeutic resistance. However, only a subset of these mechanisms has progressed into clinical testing, and randomized evidence demonstrating a meaningful survival benefit remains limited^[24,134,162,167]^.

An important distinction should therefore be made between mechanistic evidence, early clinical evidence, and randomized evidence of therapeutic benefit. Mechanistic studies can establish biological plausibility; early-phase trials can demonstrate feasibility, safety, pharmacodynamic effects, and preliminary activity; however, randomized clinical trials are required to determine whether modulation of the TME translates into meaningful improvements in patient outcomes.

The available evidence also argues against treating the PDAC TME as a uniformly detrimental structure. Different stromal populations may have context-dependent and sometimes tumor-restraining functions, and indiscriminate stromal depletion may therefore produce unintended consequences^[148,149]^.

Within this framework, the PDAC resistance ecosystem may provide a useful perspective for future therapeutic development. Rather than targeting isolated components of the TME, combination approaches could simultaneously interfere with complementary resistance mechanisms, for example, stromal signaling together with oncogenic MAPK signaling, radiotherapy together with FAK inhibition, or TME remodeling together with immune activation. The objective is not simply to eliminate the TME, but to disrupt the adaptive interactions that allow tumor cells to survive therapeutic pressure. Figure 3 highlights therapeutic strategies for targeting the PDAC resistance ecosystem.

**Figure 3:**
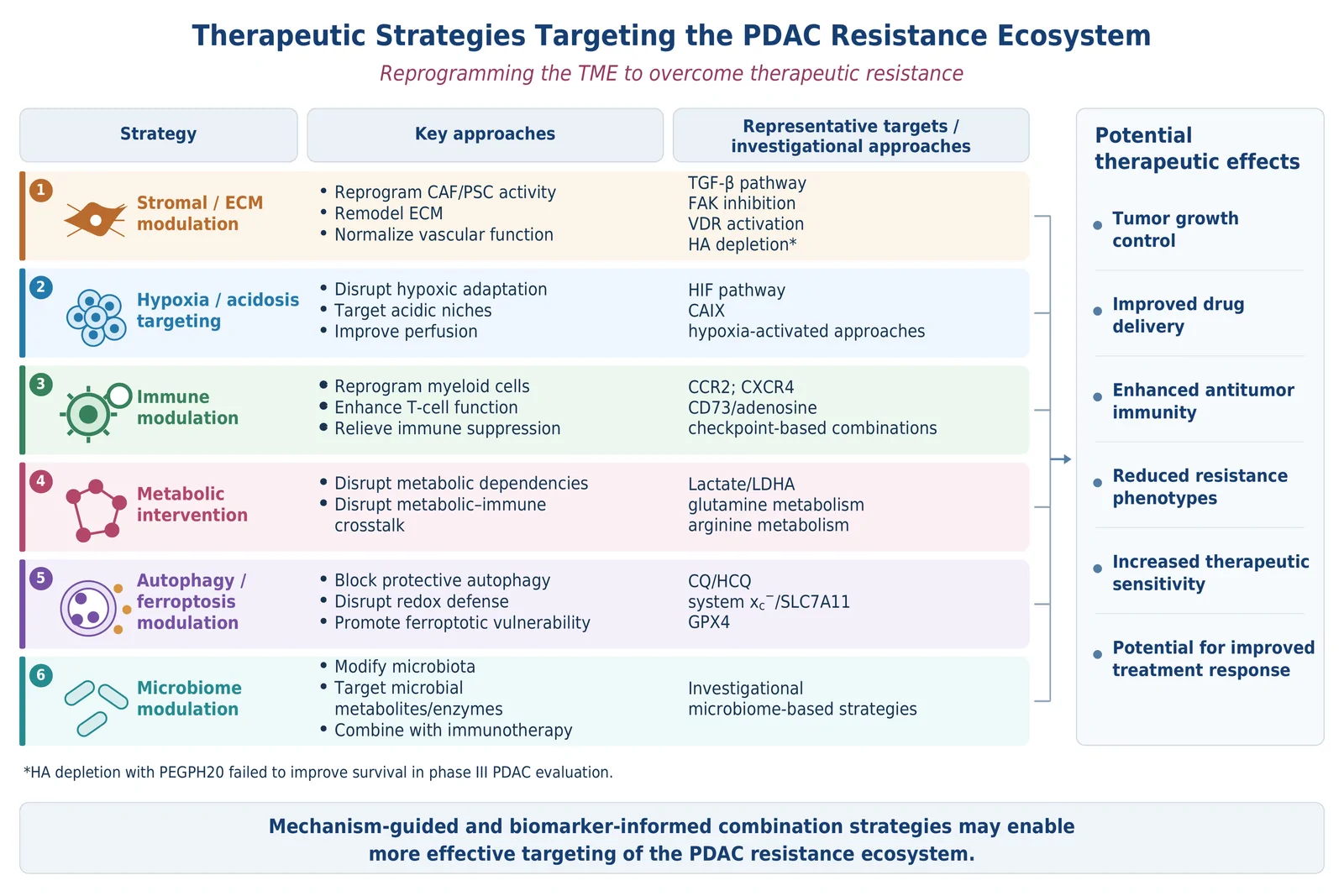
Therapeutic strategies targeting the PDAC resistance ecosystem. Therapeutic approaches directed at the PDAC resistance ecosystem aim to reprogram, rather than indiscriminately eliminate, tumor microenvironmental components and resistance-supporting processes. Stromal and ECM modulation seeks to reprogram CAF/PSC activity, remodel the ECM, and improve vascular function through pathways including TGF-β, FAK, and VDR; importantly, PEGPH20-mediated hyaluronan depletion failed to improve survival in a phase III PDAC trial, highlighting the limitations of indiscriminate stromal depletion. Additional investigational strategies include targeting hypoxia and acidosis, reprogramming immunosuppressive myeloid populations and enhancing T-cell function, disrupting metabolic dependencies and metabolic–immune crosstalk, inhibiting cytoprotective autophagy and exploiting ferroptotic vulnerability, and modulating the microbiome. These approaches may help control tumor growth, improve drug delivery, enhance antitumor immunity, reduce resistance phenotypes, and increase therapeutic sensitivity. Overall, mechanism-guided and biomarker-informed combination strategies may enable more effective targeting of the interconnected PDAC resistance ecosystem; however, many of these approaches remain investigational and should not be interpreted as having established clinical efficacy. PDAC: Pancreatic ductal adenocarcinoma; ECM: extracellular matrix; CAF: cancer-associated fibroblast; PSC: pancreatic stellate cell; TGF-β: transforming growth factor-β; FAK: focal adhesion kinase; VDR: vitamin D receptor; PEGPH20: pegylated recombinant human hyaluronidase; TME: tumor microenvironment; HA: hyaluronan; HIF: hypoxia-inducible factor; CAIX: carbonic anhydrase IX; CCR2: C-C motif chemokine receptor 2; CXCR4: C-X-C motif chemokine receptor 4; LDHA: lactate dehydrogenase A; CQ: chloroquine; HCQ: hydroxychloroquine; system Xc^-^: cystine/glutamate antiporter; SLC7A11: solute carrier family 7 member 11; GPX4: glutathione peroxidase 4.

## CONCLUSION AND FUTURE PERSPECTIVES

Therapeutic resistance in PDAC should increasingly be understood as an emergent property of a dynamic tumor ecosystem rather than as the consequence of isolated molecular defects. The clinical failure of several single-component TME interventions, exemplified by indiscriminate stromal depletion with PEGPH20, illustrates the limitations of treating one resistance mechanism in isolation. Future strategies should therefore integrate tumor-intrinsic vulnerabilities with TME states, including stromal composition, myeloid activity, metabolic and physicochemical conditions, and spatial organization. Such an ecosystem-oriented approach will require biomarker-guided patient selection and rational combination therapies designed to interrupt specific compensatory feedback loops rather than simply increasing the number of therapeutic targets.

## DECLARATIONS

### AI and AI-assisted tools statement

During the preparation of this manuscript, ChatGPT (GPT-5.6, version 5.6, released 2024-11-18) was used to assist with language editing. The AI-assisted explanatory figures were created specifically for this manuscript and do not reproduce or incorporate copyrighted figures, panels, or graphical materials from previously published works. Figures 1-3 were prepared with the assistance of OpenAI ChatGPT (GPT-6 Astra, version 6.0, released 2025-08-20) and its integrated image-generation tool for visual organization and graphical rendering. The scientific content, terminology, therapeutic strategies, labels, mechanistic relationships, and graphical representations were independently reviewed and verified by the authors for accuracy and consistency with the manuscript. The authors take full responsibility for the scientific accuracy and final content of Figures 1-3. The authors reviewed the final visual content and take responsibility for ensuring that it does not infringe third-party copyright. The final Graphical Abstract was independently recreated using BioRender in accordance with the journal’s policy; applicable licensing and attribution requirements for the software and graphical assets have been followed. The authors take full responsibility for the scientific accuracy, integrity, and final content of the manuscript.

### Copyright

© The Author(s) 2026.

## References

[1] Siegel RL, Giaquinto AN, Jemal A (2024). Cancer statistics, 2024. CA Cancer J Clin.

[2] Siegel RL, Kratzer TB, Giaquinto AN, Sung H, Jemal A (2025). Cancer statistics, 2025. CA Cancer J Clin.

[3] Rahib L, Smith BD, Aizenberg R, Rosenzweig AB, Fleshman JM, Matrisian LM (2014). Projecting cancer incidence and deaths to 2030: the unexpected burden of thyroid, liver, and pancreas cancers in the United States. Cancer Res.

[4] Koltai T, Reshkin SJ, Carvalho TMA (2022). Resistance to gemcitabine in pancreatic ductal adenocarcinoma: a physiopathologic and pharmacologic review. Cancers.

[5] Mannucci A, Goel A (2026). Advances in pancreatic cancer early diagnosis, prevention, and treatment: the past, the present, and the future. CA Cancer J Clin.

[6] Gao J, Wang J, Guan C (2024). Advances in drug therapy for metastatic pancreatic ductal adenocarcinoma. J Cancer.

[7] Singh NK, Ahmad F, Husain A (2026). Molecular dissection of pancreatic cancer signaling: toward targeted therapies for KRAS, MDM2-TP53, EGFR, and PI3K/AKT/mTOR. Pathol Res Pract.

[8] Kumar S, Singh SK, Srivastava P, Suresh S, Rana B, Rana A (2023). Interplay between MAP kinases and tumor microenvironment: opportunity for immunotherapy in pancreatic cancer. Adv Cancer Res.

[9] Ghanwatkar Y, Ramanathan R, Mahato RI (2026). Desmoplasia and therapeutic resistance in pancreatic ductal adenocarcinoma. Cancer Lett.

[10] Zhou J, Li J, Lu H, Hong Y (2025). Deciphering the secrets of tumor-initiating cells in pancreatic ductal adenocarcinoma microenvironment: mechanisms and therapeutic opportunities. Front Immunol.

[11] Sharma R, Malviya R (2023). Correlation between hypoxia and HGF/c-MET expression in the management of pancreatic cancer. Biochim Biophys Acta Rev Cancer.

[12] Suresh DS, Salcedo-Noriega MF, Vaish U (2026). Cytokine circuitry in pancreatic cancer: targets for overcoming immune checkpoint inhibitor resistance in PDAC. Cytokine Growth Factor Rev.

[13] Zhao K, Xiao MM, Yang YS, Xiao X (2025). Tumor microenvironment phenotyping guides precision therapy in unresectable pancreatic cancer. World J Gastrointest Oncol.

[14] Zhang P, Pan J, Lin S (2025). Smart drug delivery platforms reprogramming cancer immune cycle to mitigate immune resistance of pancreatic tumors. Adv Drug Deliv Rev.

[15] Sherman MH, Beatty GL (2023). Tumor microenvironment in pancreatic cancer pathogenesis and therapeutic resistance. Annu Rev Pathol.

[16] Ju Y, Xu D, Liao MM (2024). Barriers and opportunities in pancreatic cancer immunotherapy. NPJ Precis Oncol.

[17] Qi H, Lv G, Zhang S, Xue R, Liu M (2026). Unraveling pancreatic ductal adenocarcinoma at single-cell resolution with spatial insights: from mechanisms to clinical translation. Cancer Lett.

[18] Werba G, Weissinger D, Kawaler EA (2023). Single-cell RNA sequencing reveals the effects of chemotherapy on human pancreatic adenocarcinoma and its tumor microenvironment. Nat Commun.

[19] Chen MM, Gao Q, Ning H (2025). Integrated single-cell and spatial transcriptomics uncover distinct cellular subtypes involved in neural invasion in pancreatic cancer. Cancer Cell.

[20] Mao X, Xu J, Wang W (2021). Crosstalk between cancer-associated fibroblasts and immune cells in the tumor microenvironment: new findings and future perspectives. Mol Cancer.

[21] Yu X, Yang J, Xu J (2024). Histone lactylation: from tumor lactate metabolism to epigenetic regulation. Int J Biol Sci.

[22] Yan J, Ou H, Wang S, Jin K, Yu X, Wu W (2026). Overcoming multidimensional immunotherapy resistance in PDAC: from microenvironment to clinic. Front Immunol.

[23] Shi J, Cui X, Wang J, Liu G, Meng J, Zhang Y (2025). Crosstalk between the tumor immune microenvironment and metabolic reprogramming in pancreatic cancer: new frontiers in immunotherapy. Front Immunol.

[24] Kung HC, Zheng KW, Zimmerman JW, Zheng L (2025). The tumour microenvironment in pancreatic cancer - new clinical challenges, but more opportunities. Nat Rev Clin Oncol.

[25] Espona-Fiedler M, Patthey C, Lindblad S, Sarró I, Öhlund D (2024). Overcoming therapy resistance in pancreatic cancer: new insights and future directions. Biochem Pharmacol.

[26] Saúde-Conde R, Arçay Öztürk A, Stosic K (2024). Cancer-associated fibroblasts in pancreatic ductal adenocarcinoma or a metaphor for heterogeneity: from single-cell analysis to whole-body imaging. Biomedicines.

[27] Sato H, Hara T, Meng S (2023). Multifaced roles of desmoplastic reaction and fibrosis in pancreatic cancer progression: current understanding and future directions. Cancer Sci.

[28] Sigirli S, Karakas D (2025). Fibrotic fortresses and therapeutic frontiers: pancreatic stellate cells and the extracellular matrix in pancreatic cancer. Cancer Med.

[29] Liu X, Shao Y, Li Y (2025). Extensive review of nanomedicine strategies targeting the tumor microenvironment in PDAC. Int J Nanomedicine.

[30] Gong P, Wang F, Hua Y, Ying J, Chen J, Qiao Y (2025). Collagenase-mediated extracellular matrix targeting for enhanced drug penetration and therapeutic efficacy in nanoscale delivery systems for cancer therapy. J Nanobiotechnology.

[31] Wang D, Li Y, Ge H, Ghadban T, Reeh M, Güngör C (2022). The extracellular matrix: a key accomplice of cancer stem cell migration, metastasis formation, and drug resistance in PDAC. Cancers.

[32] Detert K, Piotrowsky A, Marongiu L (2026). Progress and challenges in the development of advanced pancreatic cancer organoids. J Exp Clin Cancer Res.

[33] Urbanova M, Cihova M, Buocikova V (2023). Nanomedicine and epigenetics: new alliances to increase the odds in pancreatic cancer survival. Biomed Pharmacother.

[34] Cai W, Chen Y, Dai S, Zhao J, Chen L (2025). Hyaluronic acid and its role in pancreatic cancer: mechanisms of tumor progression, drug resistance, and therapeutic targeting. Pharmacol Res.

[35] Guo Z, Ashrafizadeh M, Zhang W, Zou R, Sethi G, Zhang X (2024). Molecular profile of metastasis, cell plasticity and EMT in pancreatic cancer: a pre-clinical connection to aggressiveness and drug resistance. Cancer Metastasis Rev.

[36] Hamada S, Matsumoto R, Masamune A (2022). Pancreatic stellate cells and metabolic alteration: physiology and pathophysiology. Front Physiol.

[37] Ando R, Sakai A, Iida T, Kataoka K, Mizutani Y, Enomoto A (2022). Good and bad stroma in pancreatic cancer: relevance of functional states of cancer-associated fibroblasts. Cancers.

[38] Menezes S, Okail MH, Jalil SMA, Kocher HM, Cameron AJM (2022). Cancer-associated fibroblasts in pancreatic cancer: new subtypes, new markers, new targets. J Pathol.

[39] Zhang Q, Cao Z, Yan S (2025). Metabolic and immune crosstalk between cancer-associated fibroblasts and pancreatic cancer cells. J Transl Med.

[40] Wu J, Dai T, Li Z (2025). Integrating multi-modal transcriptomics identifies cellular subtypes with distinct roles in PDAC progression. Cell Oncol.

[41] Biffi G, Tuveson DA (2021). Diversity and biology of cancer-associated fibroblasts. Physiol Rev.

[42] Krishnamurty AT, Shyer JA, Thai M (2022). LRRC15^+^ myofibroblasts dictate the stromal setpoint to suppress tumour immunity. Nature.

[43] Zhang T, Ren Y, Yang P, Wang J, Zhou H (2022). Cancer-associated fibroblasts in pancreatic ductal adenocarcinoma. Cell Death Dis.

[44] Limbu KR, Chhetri RB, Oh YS, Oak MH, Beak DJ, Park EY (2025). Oxidative stress and pancreatic cancer: a dual role in tumorigenesis and drug toxicity. Toxicol Res.

[45] Grobbelaar C, Kgomo M, Mabeta P (2024). Angiogenesis and pancreatic cancer: novel approaches to overcome treatment resistance. Curr Cancer Drug Targets.

[46] Mosalem OM, Abdelhakeem A, Abdel-Razeq NH, Babiker H (2025). Pancreatic ductal adenocarcinoma (PDAC): clinical progress in the last five years. Expert Opin Investig Drugs.

[47] Hsu SK, Jadhao M, Liao WT, Chang WT, Hung CT, Chiu CC (2022). Culprits of PDAC resistance to gemcitabine and immune checkpoint inhibitor: tumour microenvironment components. Front Mol Biosci.

[48] Yao H, Luo L, Li R (2024). New insight into the role of SMAD4 mutation/deficiency in the prognosis and therapeutic resistance of pancreatic ductal adenocarcinomas. Biochim Biophys Acta Rev Cancer.

[49] Song N, Cui K, Zeng L (2024). Advance in the role of chemokines/chemokine receptors in carcinogenesis: focus on pancreatic cancer. Eur J Pharmacol.

[50] Xu J, Yao H, Wang J (2025). Perineural invasion and the “cold” tumor microenvironment in pancreatic cancer: mechanisms of crosstalk and therapeutic opportunities. Front Immunol.

[51] Guo X, Zhang H, Yao L, Ma W (2026). Harnessing the effector-to-regulatory T cell ratio to advance prognosis and precision therapy in pancreatic cancer. Crit Rev Oncol Hematol.

[52] Pan D, Li X, Qiao X, Wang Q (2025). Immunosuppressive tumor microenvironment in pancreatic cancer: mechanisms and therapeutic targets. Front Immunol.

[53] Kemp SB, Pasca di Magliano M, Crawford HC (2021). Myeloid cell mediated immune suppression in pancreatic cancer. Cell Mol Gastroenterol Hepatol.

[54] Jiang S, Ren B, Ding C (2025). Polyamines in pancreatic cancer: reshaping the immunosuppressive tumor microenvironment. Cancer Lett.

[55] Weizman N, Krelin Y, Shabtay-Orbach A (2014). Macrophages mediate gemcitabine resistance of pancreatic adenocarcinoma by upregulating cytidine deaminase. Oncogene.

[56] Halbrook CJ, Pontious C, Kovalenko I (2019). Macrophage-released pyrimidines inhibit gemcitabine therapy in pancreatic cancer. Cell Metab.

[57] D’Errico G, Alonso-Nocelo M, Vallespinos M (2019). Tumor-associated macrophage-secreted 14-3-3ζ signals via AXL to promote pancreatic cancer chemoresistance. Oncogene.

[58] Rohila D, Park IH, Pham TV (2023). Syk inhibition reprograms tumor-associated macrophages and overcomes gemcitabine-induced immunosuppression in pancreatic ductal adenocarcinoma. Cancer Res.

[59] Nywening TM, Belt BA, Cullinan DR (2018). Targeting both tumour-associated CXCR2^+^ neutrophils and CCR2^+^ macrophages disrupts myeloid recruitment and improves chemotherapeutic responses in pancreatic ductal adenocarcinoma. Gut.

[60] Peng L, Liang Y, Guo X (2025). Nanomedicine breakthroughs overcoming pancreatic cancer drug resistance through precision nano-interventions. Nanoscale Adv.

[61] Velasco RM, García AG, Sánchez PJ, Sellart IM, Sánchez-Arévalo Lobo VJ (2023). Tumour microenvironment and heterotypic interactions in pancreatic cancer. J Physiol Biochem.

[62] Lin HJ, Liu Y, Caroland K, Lin J (2023). Polarization of cancer-associated macrophages maneuver neoplastic attributes of pancreatic ductal adenocarcinoma. Cancers.

[63] Salehi-Sangani P, Maharati AH, Payami B, Aliakbarian M, Abbaszadegan MR (2025). HypoxamiRs in pancreatic cancer: master regulators of the hypoxic tumor microenvironment. Cell Biol Toxicol.

[64] Tao J, Yang G, Zhou W (2021). Targeting hypoxic tumor microenvironment in pancreatic cancer. J Hematol Oncol.

[65] Hao X, Ren Y, Feng M, Wang Q, Wang Y (2021). Metabolic reprogramming due to hypoxia in pancreatic cancer: implications for tumor formation, immunity, and more. Biomed Pharmacother.

[66] Gan L, Luo P, Zou J (2025). Crosstalk between hypoxia-inducible factor (HIF) and lncRNAs in digestive tumors: from molecular mechanisms to clinical translation. Front Cell Dev Biol.

[67] Toledo B, Deiana C, Scianò F (2024). Treatment resistance in pancreatic and biliary tract cancer: molecular and clinical pharmacology perspectives. Expert Rev Clin Pharmacol.

[68] Chung WC, Xu K (2023). Notch signaling pathway in pancreatic tumorigenesis. Adv Cancer Res.

[69] Palamaris K, Felekouras E, Sakellariou S (2021). Epithelial to mesenchymal transition: key regulator of pancreatic ductal adenocarcinoma progression and chemoresistance. Cancers.

[70] (2018). da Silva VP, Mesquita CB, Nunes JS, de Bem Prunes B, Rados PV, Visioli F. Effects of extracellular acidity on resistance to chemotherapy treatment: a systematic review. Med Oncol.

[71] Stigliani A, Ialchina R, Yao J (2024). Adaptation to an acid microenvironment promotes pancreatic cancer organoid growth and drug resistance. Cell Rep.

[72] Cappellesso F, Orban MP, Shirgaonkar N (2022). Targeting the bicarbonate transporter SLC4A4 overcomes immunosuppression and immunotherapy resistance in pancreatic cancer. Nat Cancer.

[73] Zhang J, Kong X, Zhou B (2025). Metabolic reprogramming of drug resistance in pancreatic cancer: mechanisms and effects. Mol Aspects Med.

[74] Zuzčák M, Trnka J (2022). Cellular metabolism in pancreatic cancer as a tool for prognosis and treatment (Review). Int J Oncol.

[75] Tuerhong A, Xu J, Shi S (2021). Overcoming chemoresistance by targeting reprogrammed metabolism: the Achilles’ heel of pancreatic ductal adenocarcinoma. Cell Mol Life Sci.

[76] Li X, Zhou J, Wang X (2023). Pancreatic cancer and fibrosis: targeting metabolic reprogramming and crosstalk of cancer-associated fibroblasts in the tumor microenvironment. Front Immunol.

[77] Zhao Z, Mei Y, Wang Z, He W (2022). The effect of oxidative phosphorylation on cancer drug resistance. Cancers.

[78] Ma J, Bhardwaj V, Lobie PE, Pandey V (2026). Metabolic plasticity in pancreatic ductal adenocarcinoma progression and response to treatment. Mol Cancer.

[79] Fang L, Gao D, Jiang Z, Li G, Li M (2025). Glutamine’s double-edged sword: fueling tumor growth and offering therapeutic hope. Front Immunol.

[80] Zhang Y, Yang Z, Liu Y, Pei J, Li R, Yang Y (2025). Targeting lipid metabolism: novel insights and therapeutic advances in pancreatic cancer treatment. Lipids Health Dis.

[81] Kalluri R, LeBleu VS (2020). The biology, function, and biomedical applications of exosomes. Science.

[82] Binenbaum Y, Fridman E, Yaari Z (2018). Transfer of miRNA in macrophage-derived exosomes induces drug resistance in pancreatic adenocarcinoma. Cancer Res.

[83] Xavier CPR, Castro I, Caires HR (2021). Chitinase 3-like-1 and fibronectin in the cargo of extracellular vesicles shed by human macrophages influence pancreatic cancer cellular response to gemcitabine. Cancer Lett.

[84] Zheng S, Tian Q, Yuan Y (2023). Extracellular vesicle-packaged circBIRC6 from cancer-associated fibroblasts induce platinum resistance via SUMOylation modulation in pancreatic cancer. J Exp Clin Cancer Res.

[85] Yu M, Su M, Tian Z (2025). Extracellular vesicle-packaged linc-ZNF25-1 from pancreatic cancer cell promotes pancreatic stellate cell uptake of asparagine to advance chemoresistance. Adv Sci.

[86] Patel GK, Khan MA, Zubair H (2019). Comparative analysis of exosome isolation methods using culture supernatant for optimum yield, purity and downstream applications. Sci Rep.

[87] Capula M, Perán M, Xu G (2022). Role of drug catabolism, modulation of oncogenic signaling and tumor microenvironment in microbe-mediated pancreatic cancer chemoresistance. Drug Resist Updat.

[88] Bautista J, Bedón-Galarza R, Martínez-Hidalgo F (2026). Decoding the microbial blueprint of pancreatic cancer. Front Med.

[89] Zheng SH, Li KZ, Feng G (2025). Gut microbiota reshaping the pancreatic cancer immune microenvironment: new avenues for immunotherapy. Mol Cancer.

[90] Guo Z, Liu Q (2026). Dual role of exosomes in pancreatic cancer: underlying mechanisms and research advances (Review). Oncol Lett.

[91] Carretero-Fernández M, Ruíz-Durán L, Uysal-Onganer P (2026). Non-coding RNAs as biomarkers and therapeutic targets in pancreatic cancer: clinical implications and translational perspectives. Biomed Pharmacother.

[92] Wu Y, Jiang X, Yu Z, Xing Z, Ma Y, Qing H (2025). Mechanisms of anti-PD therapy resistance in digestive system neoplasms. Recent Pat Anticancer Drug Discov.

[93] Liu Y, Dai Y, Wang L (2026). Spatial omics at the forefront: emerging technologies, analytical innovations, and clinical applications. Cancer Cell.

[94] Malchiodi ZX, Lekan AA, Suter RK (2026). Periductal fibroblast density defines lymphocyte exclusion via a CD44-dependent stromal checkpoint in pancreatic cancer. Cancer Immunol Res.

[95] Zhang Z, Zhang H, Liu T, Chen T, Wang D, Tang D (2021). Heterogeneous pancreatic stellate cells are powerful contributors to the malignant progression of pancreatic cancer. Front Cell Dev Biol.

[96] Hermann PC, Huber SL, Herrler T (2007). Distinct populations of cancer stem cells determine tumor growth and metastatic activity in human pancreatic cancer. Cell Stem Cell.

[97] Batlle E, Clevers H (2017). Cancer stem cells revisited. Nat Med.

[98] Li C, Heidt DG, Dalerba P (2007). Identification of pancreatic cancer stem cells. Cancer Res.

[99] Phi LTH, Sari IN, Yang YG (2018). Cancer stem cells (CSCs) in drug resistance and their therapeutic implications in cancer treatment. Stem Cells Int.

[100] Sancho P, Burgos-Ramos E, Tavera A (2015). MYC/PGC-1α balance determines the metabolic phenotype and plasticity of pancreatic cancer stem cells. Cell Metab.

[101] Yang S, Kimmelman AC (2011). A critical role for autophagy in pancreatic cancer. Autophagy.

[102] Yang A, Rajeshkumar NV, Wang X (2014). Autophagy is critical for pancreatic tumor growth and progression in tumors with p53 alterations. Cancer Discov.

[103] Rausch V, Liu L, Apel A (2012). Autophagy mediates survival of pancreatic tumour-initiating cells in a hypoxic microenvironment. J Pathol.

[104] Sousa CM, Biancur DE, Wang X (2016). Pancreatic stellate cells support tumour metabolism through autophagic alanine secretion. Nature.

[105] Kinsey CG, Camolotto SA, Boespflug AM (2019). Protective autophagy elicited by RAF→MEK→ERK inhibition suggests a treatment strategy for RAS-driven cancers. Nat Med.

[106] Nazio F, Bordi M, Cianfanelli V, Locatelli F, Cecconi F (2019). Autophagy and cancer stem cells: molecular mechanisms and therapeutic applications. Cell Death Differ.

[107] Dixon SJ, Lemberg KM, Lamprecht MR (2012). Ferroptosis: an iron-dependent form of nonapoptotic cell death. Cell.

[108] Zhang C, Liu X, Jin S, Chen Y, Guo R (2022). Ferroptosis in cancer therapy: a novel approach to reversing drug resistance. Mol Cancer.

[109] Badgley MA, Kremer DM, Maurer HC (2020). Cysteine depletion induces pancreatic tumor ferroptosis in mice. Science.

[110] Dai E, Han L, Liu J (2020). Ferroptotic damage promotes pancreatic tumorigenesis through a TMEM173/STING-dependent DNA sensor pathway. Nat Commun.

[111] Cui K, Wang K, Huang Z (2024). Ferroptosis and the tumor microenvironment. J Exp Clin Cancer Res.

[112] Lei G, Zhuang L, Gan B (2024). The roles of ferroptosis in cancer: tumor suppression, tumor microenvironment, and therapeutic interventions. Cancer Cell.

[113] Lu L, Johnson CH, Khan SA, Irwin ML (2026). GDF15 in the tumor microenvironment: a central mediator of cancer immunometabolism and therapeutic resistance. Cytokine Growth Factor Rev.

[114] Sun K, Zhang X, Shi J (2025). Elevated protein lactylation promotes immunosuppressive microenvironment and therapeutic resistance in pancreatic ductal adenocarcinoma. J Clin Invest.

[115] King RJ, Shukla SK, He C (2022). CD73 induces GM-CSF/MDSC-mediated suppression of T cells to accelerate pancreatic cancer pathogenesis. Oncogene.

[116] Tang T, Huang X, Lu M, Zhang G, Han X, Liang T (2023). Transcriptional control of pancreatic cancer immunosuppression by metabolic enzyme CD73 in a tumor-autonomous and -autocrine manner. Nat Commun.

[117] Ala M (2021). The footprint of kynurenine pathway in every cancer: a new target for chemotherapy. Eur J Pharmacol.

[118] Peng M, Ying Y, Zhang Z, Liu L, Wang W (2023). Reshaping the pancreatic cancer microenvironment at different stages with chemotherapy. Cancers.

[119] Bruciamacchie M, Garambois V, Vie N (2025). ATR inhibition potentiates FOLFIRINOX cytotoxic effect in models of pancreatic ductal adenocarcinoma by remodelling the tumour microenvironment. Br J Cancer.

[120] Liu X, Baer JM, Stone ML (2024). Stromal reprogramming overcomes resistance to RAS-MAPK inhibition to improve pancreas cancer responses to cytotoxic and immune therapy. Sci Transl Med.

[121] Lian M, Mortoglou M, Uysal-Onganer P (2023). Impact of hypoxia-induced miR-210 on pancreatic cancer. Curr Issues Mol Biol.

[122] Ala M (2024). Noncoding ribonucleic acids (RNAs) may improve response to immunotherapy in pancreatic cancer. ACS Pharmacol Transl Sci.

[123] Otieno MO, Powrózek T, Garcia-Foncillas J, Martinez-Useros J (2025). The crosstalk within tumor microenvironment and exosomes in pancreatic cancer. Biochim Biophys Acta Rev Cancer.

[124] Kumarasamy V, Wang J, Frangou C (2024). The extracellular niche and tumor microenvironment enhance KRAS inhibitor efficacy in pancreatic cancer. Cancer Res.

[125] Ye X, Yu Y, Zheng X, Ma H (2024). Clinical immunotherapy in pancreatic cancer. Cancer Immunol Immunother.

[126] Liu J, Zhang B, Huang B (2024). A stumbling block in pancreatic cancer treatment: drug resistance signaling networks. Front Cell Dev Biol.

[127] Tao X, Go V, Xiao GG (2021). Progress on the role of reactive oxygen species-mediated tumor microenvironment in pancreatic cancer. Acta Physiol Sin.

[128] Jang JE, Hajdu CH, Liot C, Miller G, Dustin ML, Bar-Sagi D (2017). Crosstalk between regulatory T cells and tumor-associated dendritic cells negates anti-tumor immunity in pancreatic cancer. Cell Rep.

[129] Ho WJ, Jaffee EM, Zheng L (2020). The tumour microenvironment in pancreatic cancer - clinical challenges and opportunities. Nat Rev Clin Oncol.

[130] Barker HE, Paget JT, Khan AA, Harrington KJ (2015). The tumour microenvironment after radiotherapy: mechanisms of resistance and recurrence. Nat Rev Cancer.

[131] Jing X, Yang F, Shao C (2019). Role of hypoxia in cancer therapy by regulating the tumor microenvironment. Mol Cancer.

[132] Rankin EB, Giaccia AJ (2016). Hypoxic control of metastasis. Science.

[133] Horsman MR, Mortensen LS, Petersen JB, Busk M, Overgaard J (2012). Imaging hypoxia to improve radiotherapy outcome. Nat Rev Clin Oncol.

[134] Lander VE, Belle JI, Kingston NL (2022). Stromal reprogramming by FAK inhibition overcomes radiation resistance to allow for immune priming and response to checkpoint blockade. Cancer Discov.

[135] Nair ST, Abhi C, Kamalasanan K (2024). Pathophysiology-driven approaches for overcoming nanomedicine resistance in pancreatic cancer. Mol Pharm.

[136] (2020). Van Cutsem E, Tempero MA, Sigal D, et al.; HALO 109-301 Investigators. Randomized phase III trial of pegvorhyaluronidase alfa with nab-paclitaxel plus gemcitabine for patients with hyaluronan-high metastatic pancreatic adenocarcinoma.. J Clin Oncol.

[137] Perez KJ, Dias Costa A, Jordan A (2026). Gemcitabine and nab-paclitaxel with or without the VDR agonist paricalcitol for metastatic pancreatic cancer: a randomized, multiarm, run-in phase trial. Nat Cancer.

[138] Easty D, McDermott R, Murphy AG (2026). Early termination of NCT04617067, a phase II, open label, clinical trial of oral paricalcitol in combination with gemcitabine and NAB-paclitaxel therapy in advanced pancreatic cancer. BMC Cancer.

[139] Nywening TM, Wang-Gillam A, Sanford DE (2016). Targeting tumour-associated macrophages with CCR2 inhibition in combination with FOLFIRINOX in patients with borderline resectable and locally advanced pancreatic cancer: a single-centre, open-label, dose-finding, non-randomised, phase 1b trial. Lancet Oncol.

[140] Noel M, O’Reilly EM, Wolpin BM (2020). Phase 1b study of a small molecule antagonist of human chemokine (C-C motif) receptor 2 (PF-04136309) in combination with nab-paclitaxel/gemcitabine in first-line treatment of metastatic pancreatic ductal adenocarcinoma. Invest New Drugs.

[141] Bever KM, Shin SM, Durham JN (2025). A phase 2 trial of CXCR4 antagonism and PD1 inhibition in metastatic pancreatic adenocarcinoma reveals recruitment of T cells but also immunosuppressive macrophages. Oncoimmunology.

[142] Coveler AL, Reilley MJ, Zalupski M (2024). A phase Ib/II randomized clinical trial of oleclumab with or without durvalumab plus chemotherapy in patients with metastatic pancreatic ductal adenocarcinoma. Clin Cancer Res.

[143] Beatty GL, Chiorean EG, Fishman MP (2011). CD40 agonists alter tumor stroma and show efficacy against pancreatic carcinoma in mice and humans. Science.

[144] Strickler JH, Satake H, George TJ (2023). Sotorasib in KRAS p.G12C-mutated advanced pancreatic cancer. N Engl J Med.

[145] Mahadevan KK, McAndrews KM, LeBleu VS (2023). KRAS^G12D^ inhibition reprograms the microenvironment of early and advanced pancreatic cancer to promote FAS-mediated killing by CD8^+^ T cells. Cancer Cell.

[146] Provenzano PP, Cuevas C, Chang AE, Goel VK, Von Hoff DD, Hingorani SR (2012). Enzymatic targeting of the stroma ablates physical barriers to treatment of pancreatic ductal adenocarcinoma. Cancer Cell.

[147] Sherman MH, Yu RT, Engle DD (2014). Vitamin D receptor-mediated stromal reprogramming suppresses pancreatitis and enhances pancreatic cancer therapy. Cell.

[148] Özdemir BC, Pentcheva-Hoang T, Carstens JL (2014). Depletion of carcinoma-associated fibroblasts and fibrosis induces immunosuppression and accelerates pancreas cancer with reduced survival. Cancer Cell.

[149] Rhim AD, Oberstein PE, Thomas DH (2014). Stromal elements act to restrain, rather than support, pancreatic ductal adenocarcinoma. Cancer Cell.

[150] Johansen AM, Forsythe SD, McGrath CT (2024). TGFβ in pancreas and colorectal cancer: opportunities to overcome therapeutic resistance. Clin Cancer Res.

[151] Pothula SP, Xu Z, Goldstein D, Pirola RC, Wilson JS, Apte MV (2020). Targeting HGF/c-MET axis in pancreatic cancer. Int J Mol Sci.

[152] Melisi D, Oh DY, Hollebecque A (2021). Safety and activity of the TGFβ receptor I kinase inhibitor galunisertib plus the anti-PD-L1 antibody durvalumab in metastatic pancreatic cancer. J Immunother Cancer.

[153] Lorestani P, Dashti M, Nejati N (2024). The complex role of macrophages in pancreatic cancer tumor microenvironment: a review on cancer progression and potential therapeutic targets. Discov Oncol.

[154] Liang L, Wang W (2026). The impact of p53 mutation on tumor immune evasion: mechanistic insights and clinical implications. Front Immunol.

[155] Xu M, Zhao X, Wen T, Qu X (2024). Unveiling the role of KRAS in tumor immune microenvironment. Biomed Pharmacother.

[156] Liu X, Ding Q, Zhang H, Zhang X, Chen Q, Weng S (2025). The CD39-CD73-adenosine axis: master regulator of immune evasion and therapeutic target in pancreatic ductal adenocarcinoma. Biochim Biophys Acta Rev Cancer.

[157] Abou Khouzam R, Lehn JM, Mayr H (2023). Hypoxia, a targetable culprit to counter pancreatic cancer resistance to therapy. Cancers.

[158] McDonald PC, Winum JY, Supuran CT, Dedhar S (2012). Recent developments in targeting carbonic anhydrase IX for cancer therapeutics. Oncotarget.

[159] Borad MJ, Reddy SG, Bahary N (2015). Randomized phase II trial of gemcitabine plus TH-302 versus gemcitabine in patients with advanced pancreatic cancer. J Clin Oncol.

[160] Mayer A, Vaupel P (2013). Hypoxia, lactate accumulation, and acidosis: siblings or accomplices driving tumor progression and resistance to therapy?. Adv Exp Med Biol.

[161] Guillaumond F, Leca J, Olivares O (2013). Strengthened glycolysis under hypoxia supports tumor symbiosis and hexosamine biosynthesis in pancreatic adenocarcinoma. Proc Natl Acad Sci U S A.

[162] Beatty GL, Werba G, Lyssiotis CA, Simeone DM (2021). The biological underpinnings of therapeutic resistance in pancreatic cancer. Genes Dev.

[163] https://clinicaltrials.gov/study/NCT05669482.

[164] Lim KH, Spencer KR, Safyan RA (2024). Avutometinib/defactinib and gemcitabine/nab-paclitaxel combination in first-line metastatic pancreatic ductal adenocarcinoma: initial safety and efficacy of phase 1b/2 study (RAMP 205). J Clin Oncol.

[165] Phase II study of stereotactic body radiotherapy and focal adhesion kinase inhibitor in advanced pancreas adenocarcinoma https://clinicaltrials.gov/study/NCT04331041.

[166] Jacobetz MA, Chan DS, Neesse A (2013). Hyaluronan impairs vascular function and drug delivery in a mouse model of pancreatic cancer. Gut.

[167] Hingorani SR, Zheng L, Bullock AJ (2018). HALO 202: Randomized phase II study of PEGPH20 plus nab-paclitaxel/gemcitabine versus nab-paclitaxel/gemcitabine in patients with untreated, metastatic pancreatic ductal adenocarcinoma. J Clin Oncol.

